# Status of *Exosphaeroma amplicauda* (Stimpson, 1857), *E. aphrodita* (Boone, 1923) and description of three new species (Crustacea, Isopoda, Sphaeromatidae) from the north-eastern Pacific

**DOI:** 10.3897/zookeys.504.8049

**Published:** 2015-05-18

**Authors:** Adam R. Wall, Niel L. Bruce, Regina Wetzer

**Affiliations:** 1 Research and Collections Branch, Natural History Museum of Los Angeles County, 900 Exposition Boulevard, Los Angeles, California 90007 USA; 2 Museum of Tropical Queensland and School of Marine and Tropical Biology, James Cook University; 70–102 Flinders Street, Townsville, 4810 Australia; 3 Water Research Group (Ecology), Unit for Environmental Sciences and Management, North West University, Potchefstroom, 2520, South Africa

**Keywords:** Isopoda, Sphaeromatidae, *Exosphaeroma*, Alaska, Washington, California, intertidal

## Abstract

*Exosphaeroma amplicauda* (Stimpson, 1857) from the west coast of North America is reviewed and redescribed and revealed to be a group of closely related species. A neotype is designated and the species redescribed based on the neotype and topotypic specimens. *Exosphaeroma amplicauda* is known only from the coast of California, at Marin, Sonoma and San Mateo Counties. *Exosphaeroma aphrodita* (Boone, 1923), type locality La Jolla, California and previously considered *nomen dubium* is taken out of synonymy and re-validated. A further three species: *Exosphaeroma paydenae* **sp. n.**, *Exosphaeroma russellhansoni* **sp. n.**, and *Exosphaeroma pentcheffi* **sp. n.** are described herein. *Sphaeroma octonctum* Richardson, 1899 is placed into junior synonymy with *Exosphaeroma amplicauda*. A key to the Pacific West Coast *Exosphaeroma* is provided.

## Introduction

The Sphaeromatidae is a large family, currently with 99 accepted genera (WoRMS, World Register of Marine Species, Bruce and Schotte 2013) and nearly 700 species. The phylogenetic relationships of the Sphaeromatidea were reviewed by Wetzer et al. 2013, but no family-wide treatment since the time of Hansen (1905) and the much later key of Harrison and Ellis (1991) are available. The most recent comprehensive treatment for the United States is Richardson’s (1905) monograph, which to a degree was updated by Kensley and Schotte (1989). The number of described species and genera of North American Sphaeromatidae have slowly increased over the 20^th^ century but many species remain poorly known and attributed to inappropriate genera. At last count, marine and freshwater sphaeromatids in North America included 21 genera with a total of 67 species (seven *species inquirenda*, *incertae sedis* or both).

The North American western coast lies within the East Pacific biogeographic zone, and the Sphaeromatidae are represented by 37 species in 11 genera, six of these regarded as *species inquirenda* and *incertae sedis* (see Appendix 1). While some western coast United States species have been described in detail (e.g. Bruce and Wetzer 2004; Carvacho and Haasmann 1984; Espinosa-Pérez and Hendrickx 2002; Hendrickx and Espinosa-Pérez 1998; Wetzer and Bruce 2007) many others remain poorly described, and unrecognizable by modern standards (see Appendix 1).

One such poorly-known North American species is *Exosphaeroma amplicauda* (Stimpson, 1857). The original description of *Exosphaeroma amplicauda* is brief with a single postage-stamp sized (1.5×2.0 cm) figure of the dorsum taken from specimens “found adhering to fragments of star-fishes picked up on the beach of Tomales Bay by Mr. Samuels, 6.4 mm long and deposited at the Smithsonian” (Stimpson 1857). Stimpson (1858) later provided a paragraph-long description without additional details. The species was redescribed by Kussakin (1979) based on material collected from Amchitka Island, Alaska, some 2000 kilometers north of the type locality. Differences between Kussakin’s (1979) description and fresh material of what appeared to be *Exosphaeroma amplicauda* from California, including the type location, prompted a re-evaluation of the species. Reviewing morphological and molecular data, we realize that there is a ‘species flock’ of five morphologically similar species on the western coast of North America. Such ‘species flocks’ have been reported for other sphaeromatid genera (e.g. *Paracassidina* – see Bruce 1994; *Oxinasphaera* – see Bruce 1997) and other families (e.g. Cymothoidae, see Bruce 1986; Cirolanidae, see Bruce 2004; Aegidae, see Bruce 2004, 2009; Serolidae, see Poore 1987), but this is the first such example in the East Pacific.

We redescribe *Exosphaeroma amplicauda* from the type locality Tomales Bay (central California coast) and *Exosphaeroma aphrodita* from San Diego, and describe three new closely related species: *Exosphaeroma paydenae* sp. n., Aleutians; *Exosphaeroma russellhansoni* sp. n., Puget Sound, *Exosphaeroma pentcheffi* sp. n., Palos Verdes Peninsula.

### Abbreviations

LACM–Natural History Museum of Los Angeles County; USNM–United States National Museum, Smithsonian Institution; BM–British Museum; MCZ–Museum of Comparative Zoology Harvard; ANSP–Academy of Natural Sciences Philadelphia; UAF–University of Alaska, Fairbanks; AM–Amherst College, Massachusetts; PM–Yale Peabody Museum, Connecticut; RS–robust seta/e; PMS–plumose marginal setae; SEM–scanning electron microscopy; SCAMIT–Southern California Association of Marine Invertebrate Taxonomists. Latitudes and longitudes denoted with “~” are approximate and estimated from Google Earth.

## Material and methods

Descriptions are based on the male holotype, female allotype, and topotypic paratypes. Specimens examined have been assigned a USNM or LACM catalog/type numbers. Numbers preceded by “RW” are field and station numbers. Species descriptions were prepared using DELTA (Dallwitz et al. 1997). Setal terminology broadly follows Watling (1989).

Specimens prepared for SEM were cleaned for 10–20 seconds in a Branson 1200 ultrasonic cleaner in a weak solution of Branson GP jewelry soap and distilled water. Specimens were then dehydrated with 100% ethanol. Specimens were placed in solutions of pure ethanol and distilled water in the ratios 2:1, 1:1, 1:2, and finally into 100% ethanol (20 minutes per treatment). Once dehydrated and in 100% ethanol, hexamethyldisilzane (HMDS) was used to replace the ethanol in the specimens. Specimens were transferred through ethanol and HMDS solutions in the following ratios 2:1, 1:1, 1:2 and finally into 100% HMDS (20 minutes per treatment). Specimens were transferred from the final 100% HMDS to fresh HMDS and allowed to evaporate overnight. Specimens were mounted on carbon conductive tabs and coated with gold/palladium using an Emitech K550x sputter coater (Quorum Technologies, LTD, Kent, UK) and imaged using a Hitachi S-3000N variable pressure SEM (Hitachi, Troy, MI) at the LACM.

Drawings were made with the aid of a camera lucida and illustrations were electronically “inked” with Adobe Illustrator CS6. Whole body illustrations were made with a Wild M5D stereo dissecting scope. Appendages were illustrated by dissecting off the appendage and placing them in glycerol on a depression slide and then imaged using a Nikon Labophot-2 compound scope.

Specimens were measured by tracing their dorsal surface along their longitudinal axis with the aid of a camera lucida. A scale bar in the same plane as the specimens allowed calculation of total body length. All lengths reported were mesured in this fashion and may slightly overestimate total body length because pereonites and pleonites are expanded in this position. The lengths given in the “Material Examined” are of the largest specimen of each species and sex. Not all specimens were measured. If a length is provided and multiple specimens were present in a lot, the length refers to largest specimen. In all species mature males appear larger than females, but body lengths for mature adults are similar. Males in all species have much broader uropods than females, which contributes to this illusion. Large sexually mature males tend to be rare compared to females and subadults. Gravid females are rare. Smaller non-gravid individuals cannot be sexed. Females of the different species are virtually indistinguishable and cannot be confidently assigned to a species without an accompanying male. It appears that the largest males guard harems. No individual male-female mate guarding was observed (as occurs in *Exosphaeroma inornata* Dow, 1958 which also occurs on the Pacific west coast). All species described herein occur in aggregates either under rocks or amongst dead barnacle tests.

We provide dorsal and lateral line drawings of all males for each species. We also provide dorsal and lateral SEMs of both males and females of each species.

## Taxonomy

### Key to the north-eastern Pacific species of *Exosphaeroma* of the North American West Coast

**Figure 1.**
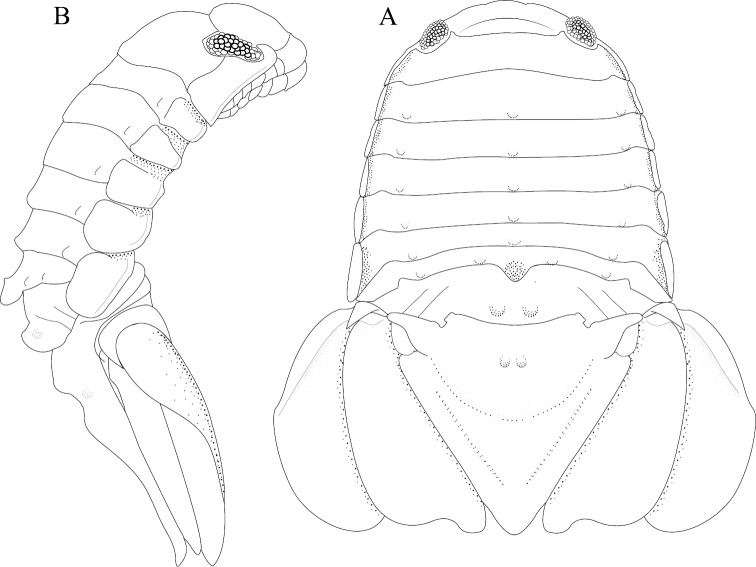
*Exosphaeroma amplicauda* male neotype LACM CR-2014.1. **A** dorsal **B** lateral.

This key is based on adult ♂ characters. Also note that weak pereon tubercles are visible only with SEM and not necessarily evident with light microscopy – e.g., compare Figures 1 and 21.

**Table:** 

1	Pereonites 1–7, pleon, and pleotelson without ornamentation; pleotelson to overall body length ratio 0.21; apex of posterior margin of pleotelson rounded and truncate; uropodal endopods posterior margin evenly rounded; sex ratio nearly 1:1; individual mate guarding	***Exosphaeroma inornata*** (Fig. 26)
–	Pleon with tubercles; pleotelson and uropods long, pleotelson to overall body length ratio 0.30 or greater; posterior margin of pleotelson acuminate; uropodal endopods posterior margin falcate; large adult males rare, one alpha male guarding many females and juveniles (harem guarding)	**2**
2	Pereonites 5–7 without tubercles; pleon with 1 anterior and 1 posterior weak tubercles on either side of longitudinal axis; pleotelson dorsal surface without ornamentation; appendix masculina straight, distally narrowing, distal apex acute, length 16.0 basal width	***Exosphaeroma paydenae* sp. n.** (Figs 5; 8B; 22)
–	Pereonite 7 with weak or strong median process; pleon with 1 medium tubercle on either side of longitudinal axis; pleotelson dorsal surface with tubercle	**3**
3	Pereonites 5 and 6 without ornamentation, pereonite 7 with weak median process; pleon with 1 medium tubercle on either side of longitudinal axis; pleotelson dorsal surface with 2 small anterior tubercles; appendix masculina distal end curving mesially, apex weakly hooked mesially, length 11.4 basal width	***Exosphaeroma russellhansoni* sp. n.** (Figs 9; 12B; 23)
–	Pereonite 5 without ornamentation, pereonite 6 with 1 lateral weak tubercle, pereonite 7 with weak median process, and paired weak lateral tubercles; pleon with 1 medium tubercle on either side of longitudinal axis; pleotelson dorsal surface with 1 anterior median strong tubercle and 2 weak medial tubercles; appendix masculina apically narrowly rounded, length 13.0 basal width	***Exosphaeroma aphrodita*** (Figs 17; 20B; 25)
–	Pereonites 5 and 6 with tubercles; pereonite 7 with median process, and tubercles; pleon with 1 posterior strong tubercle, on either side of longitudinal axis; pleotelson dorsal surface with tubercles	**4**
4	Pereonites 5 and 6 with 1 median weak tubercle, and 1 weak lateral tubercle; pereonite 7 with weak median process and paired lateral tubercles; pleotelson dorsal surface with 2 small anterior tubercles; appendix masculina distal end curving mesially, straightening at distal tip, length 15.4 basal width	***Exosphaeroma amplicauda*** (Figs 1; 4B; 21)
–	Pereonites 5–6 each with 7 longitudinal rows of strong tubercles, pereonite 7 with strong median process with 3 lateral tubercles; pleotelson dorsal surface with 3 strong medial tubercles on either side of the longitudinal axis, with 1 strong medial tubercle between the longitudinal axis and lateral margin, pleotelson covered with numerous, additional, small tubercles; appendix masculina distally narrowing to an acute rounded tip, length 15 basal width	***Exosphaeroma pentcheffi* sp. n.** (Figs 13; 16B; 24)

### Exosphaeroma

Taxon classificationAnimaliaIsopodaSphaeromatidae

Stebbing, 1900

Exosphaeroma Stebbing, 1900: 553, Restricted synonymy. – Bruce 2003: 327.

#### Type species.

*Sphaeroma gigas* Leach, 1818; by original designation (Stebbing 1900).

#### Remarks.

A diagnosis and comprehensive synonymy was provided by Bruce (2003). The genus occurs in shallow water in all the world oceans and is one of the few sphaeromatid genera extending to southern reaches of the Southern Ocean. Greatest diversity is found in the Southern Hemisphere. The genus has groups of morphologically similar species, including those species close to the type species, and a group of species with a broad rim to the pleotelson ventral margin, while some species have broad uropods and a posteriorly produced pleotelson apex. At present, the relationships between these different species groups remains unassessed.

*Exosphaeroma amplicauda* (Stimpson, 1857), *Exosphaeroma aphrodita* and the three new species described herein form a distinct group within the genus *Exosphaeroma*. This group of species is characterised by a posteriorly produced and somewhat posteriorly depressed pleotelson, with an acute apex, flattened ventrolateral margins, and the posterior margin overriding a shallow exit channel; the uropods are distally wide and the exopod is distally broadly falcate. The dorsum varies from smooth to nodular. Typically mature males of the “*amplicauda* group” have a large pleotelson and enlarged posterior coxal plates and cannot completely roll up or fold. Some similar species are known from the Southern Hemisphere, including *Exosphaeroma alveola* Bruce, 2003 (southeastern Australia); *Exosphaeroma antikraussi* Barnard, 1940, *Exosphaeroma kraussi* Tattersall, 1913, *Exosphaeroma planum* Barnard, 1914 and *Exosphaeroma varicolor* Barnard, 1914 (all South Africa); and *Exosphaeroma montis* (Hurley & Jansen, 1977) (New Zealand). All other North American *Exosphaeroma* have an evenly rounded pleotelson, with a narrow ventral margin, and uropods that are not posteriorly wide.

Other *Exosphaeroma* occurring between Alaska and the Mexican border that are morphologically not closely related to the Pacific west coast species include *Exosphaeroma inornata* (known from Puget Sound, Washington to central-southern Baja California Norte, Mexico). *Exosphaeroma inornata* differs from the “*amplicauda* group” in that *Exosphaeroma inornata* lacks marked sexual dimorphism. Males mate guard individual females with males clasping and holding females until mating. *Exosphaeroma inornata* can roll up into perfect balls, and their bodies are unornamented. This distinguishes them clearly from the “*amplicauda*” clade (*Exosphaeroma amplicauda*, *Exosphaeroma aphrodita*, and the three new species described here).

The type specimens of *Exosphaeroma rhomburum* (USNM 22573) were borrowed and consist of two specimens from Monterey Bay, neither specimen is an adult male. Richardson’s (1899b: 835) original species description only figures the pleotelson, and she did not note whether the description was based on a male or female. We were not able to further evaluate the status of this species.

In collections from the type locality at Tomales Bay in 2009 for *Exosphaeroma amplicauda*, we found “family groups” with all life stages (gravid and non-gravid females, subadult males, juveniles, and adult males). These family groups consisted of ca. 10–30 individuals, but in which adult, fully mature males are rare, leading us to conclude that males in these species guard harems rather than guard individual females. For every 10 individuals, we found one, sometimes two, large adult males. We found no evidence for multiple male morphs in these collections [e.g., alpha, beta, gamma males in *Paracerceis sculpta* as described by Shuster (1989)].

### Exosphaeroma amplicauda

Taxon classificationAnimaliaIsopodaSphaeromatidae

(Stimpson, 1857)

**Figure 2.**
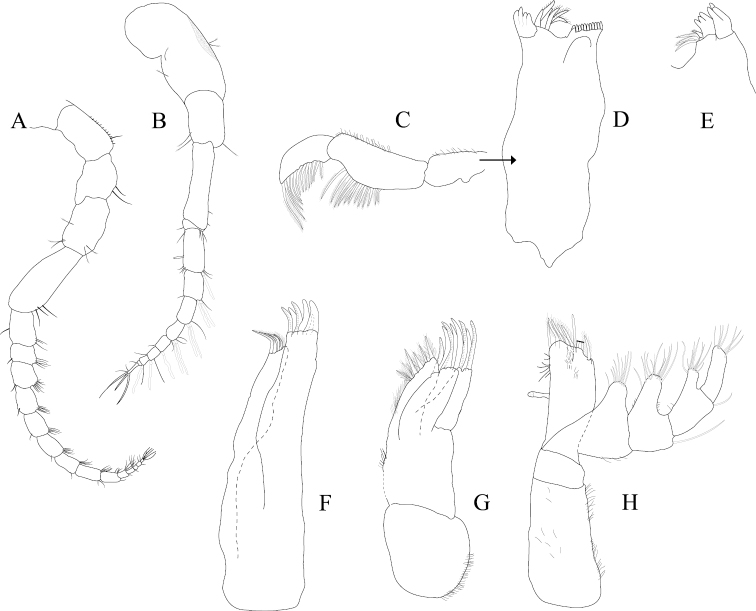
*Exosphaeroma amplicauda* male neotype LACM CR-2014.1. **A** left antenna **B** left antennula, basal article broken **C** right mandible palp **D** right mandible **E** left mandible **F** left maxillula **G** left maxilla **H** left maxilliped.

**Figure 3.**
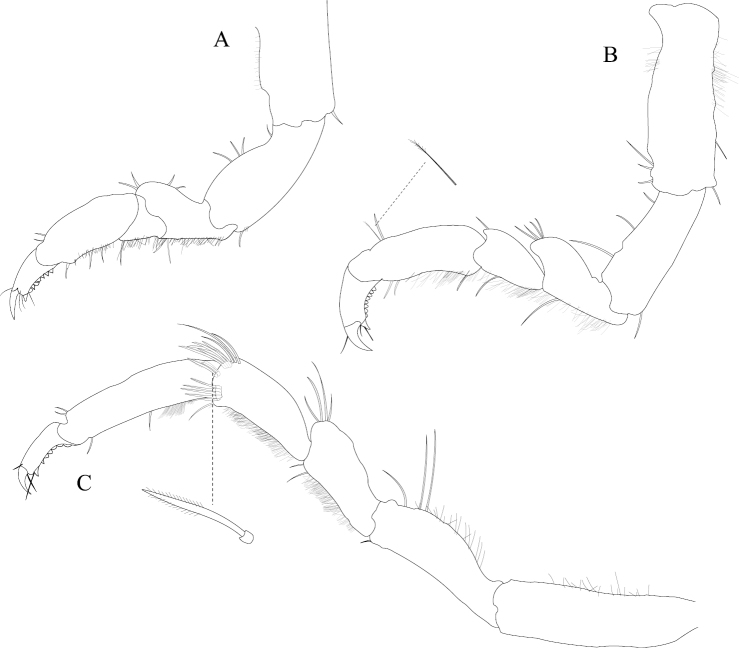
*Exosphaeroma amplicauda* male neotype LACM CR-2014.1. **A** left pereopod 1 **B** left pereopod 3 **C** left pereopod 7.

**Figure 4.**
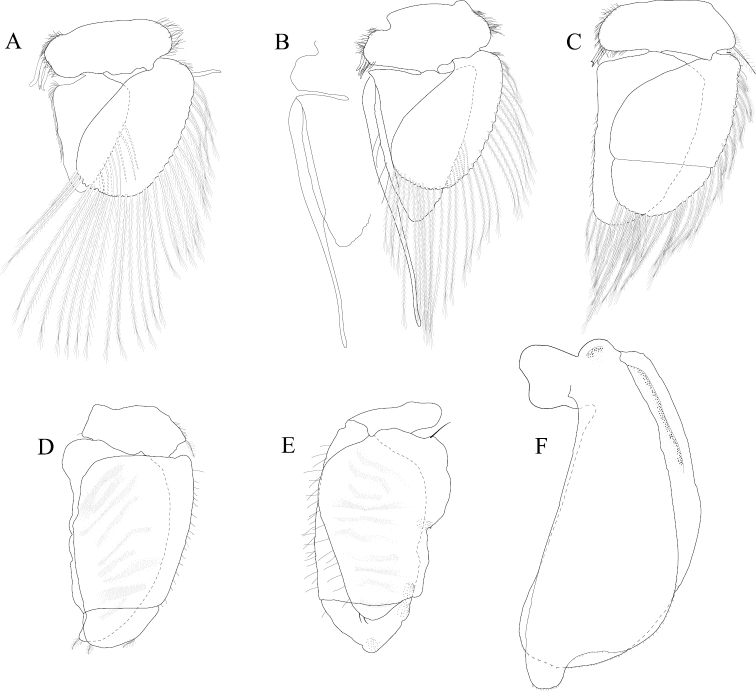
*Exosphaeroma amplicauda* male neotype LACM CR-2014.1. **A–E** left pleopods 1–5, respectively **F** right uropod.

Figures 1 , 2 , 3 , 4 , 21 , 27 , 28

Sphaeroma amplicauda Stimpson, 1857: 510; Richardson 1899a: 835; 1899b: 179; 1900: 222.
Exosphaeroma amplicauda . – Richardson 1905: 288, 289, fig. 301, 302; Gurjanova 1936: 122, fig. 69; Schultz 1969: 131, fig. 190.
Sphaeroma octoncum Richardson 1905: 293.
Exosphaeroma octoncum . – Iverson 1974: 166. – Richardson 1905: 293, fig. 309, 310. – Schotte 2012: online.
Not Exosphaeroma amplicauda . – Gurjanova 1936: 122, fig. 69. – Kussakin 1979: 399, figs 254, 255 [= *Exosphaeroma paydenae* sp. n.].

#### Material examined.

NEOTYPE (here designated): ♂ (5.1 mm): California, Marin County, Tomales Bay, north end of bay across from Hog Island, boat launch parking lot, 38.201°N, 122.922°W, intertidal, from underside of rocks, fixed and preserved in 95% ethanol, 9 Jan 2009, coll. R. Wetzer & A. Wall. RW09.003.1, LACM CR-2014.1.

**Non-type material.** 2 ♂ (RW09.003.2, LACM CR-2014.1), 3 ♀ (RW09.003.3, LACM CR-2014.1) [used for SEM], 1 ♂, ~40 ♀ and juveniles: same locality as RW09.003, LACM CR-2014.1. 6 ♂ (8.1 mm), 4 ♀ (7.1 mm), 10 juveniles (RW09.004.1), plus 2 ♂ and 4 ♀ prepared as SEM (RW09.004.2): intertidal, from underside of rocks, “family group”, coll. A. Wall. RW09.004, LACM CR-2014.2. 7 ♂ (5.8 mm), ~20 ♀ (6.8 mm) and juveniles, and 3 ♀ used for SEM: intertidal, from underside of rocks, “family group”, coll. A. Wall. RW09.005, LACM CR-2014.3. 2 ♂ (8.4 mm), ~25 ♀ (7.4 mm), and juveniles: intertidal, from empty *Balanus glandula* shells, coll. N.D. Pentcheff, RW09.006.1, LACM CR-2014.4. 1 ♂ (7.7 mm), 2 ♀ (6.5 mm), and 2 juveniles: E. side in cove across from Hog Island (Nick’s Cove), ~38.197°N, ~122.935°W, 1 Nov 1971, A.0030, coll. E.W. Iverson & J. Carlton. RW04.020.1, LACM CR-2014.5. California, Monterey County, Monterey Bay, 4 specimens (labeled *Exosphaeroma octoncum*), all are ♀. Acc. No. 03472, USNM 22574 (part).

#### Description of male.

*Body* length 1.6 width; pereonites 5–6 each with 1 median weak tubercle, and 1 weak lateral tubercle; pereonite 7 with weak median process and paired lateral tubercles (Figures 1A, B; 21A, D). *Pleon* with 1 posterior strong tubercle on either side of longitudinal axis (Figures 1A, B; 21A, D). *Pleotelson* length 0.82 width, dorsal surface with 2 small anterior tubercles; ventrolateral ridge extending posteriorly 0.75 of total length, with long setae (Figures 1A, B; 21A, C, D).

*Antennula* peduncle article 1 length 1.7 width, anterior medial margin with 2 palm setae; article 2 length 1.4 width, inferior distal margin with 2 palm setae; article 3 length 3.2 width; flagellum with 9 articles (Figure 2B). *Antenna* reaching anterior margin of pereonite 2; flagellum with 14 articles (Figure 2A).

*Left mandible* incisor with 3 cusps; lacinia mobilis with 3 cusps; lacinia mobilis spine row comprised of 5 curved, serrate spines (Figures 2E, 27D). *Right mandible* incisor with 4 cusps; spine row comprised of 6 curved, serrate spines; crushing surfaces strongly ridged (Figures 2D, 27D). *Maxillula* mesial lobe with 4 circumplumose RS; lateral lobe with 6 long, curved, pectinate RS (Figures 2F; 27E, F, G). *Maxilla* mesial lobe with 6 plumose RS on gnathal surface; middle lobe with 4 long, curved, pectinate RS; lateral lobe with 4 long, curved, pectinate RS (Figures 2G; 27E, G). *Maxilliped* endite distal surface with 5 plumose setae, and 2 simple RS; distomesial margin with 1 coupling hook; palp article 2 distal apex with 9 long, simple RS; article 3 distal apex with 12 long, simple RS, lateral distal angle with 1 long, simple RS; article 4 distal apex with 9 long, simple RS, lateral distal angle with 1 long, simple RS; article 5 distal apex with 7 long, simple RS (Figures 2H; 27E, G).

*Pereopod 1* (Figure 3A) *basis* superior margin without palm setae, inferior distal angle with 1 long, simple seta, inferior medial margin setal patch absent; *ischium* length 2.4 width, superior margin with 4 long, simple setae, inferior distal angle with 1 long, simple seta; *merus* 0.42 ischium length, superior distal angle with 2 long, simple setae; *carpus* inferior distal angle with 1 long, simple seta; *propodus* length 2.5 width, 0.82 ischium length, superior distal angle with 2 long, simple setae, inferior margin with 3 long, simple setae; *dactylus* length 1.7 width, length 0.33 propodus length, inferior margin covered with scales, distal margin with 4 simple setae (Figure 3A). *Pereopod 3* (Figures 3B, 27A) *basis* superior margin without palm setae, inferior distal angle with 1 long simple seta, inferior proximal margin with setal patch present; *ischium* length 2.5 width, superior margin with 3 long, simple RS, inferior distal angle with 1 simple RS, and with setal patch absent; *merus* lobate, length 1.4 width, 0.57 ischium length, superior distal angle with cluster of 4 simple RS, inferior margin covered in setal mat; *carpus* length 0.71 merus length, 1.2 width, superior margin with 1 long, simple seta on distal angle, inferior margin with setal mat and 1 long, simple seta; *propodus* weakly curved, length 2.5 width, 2.3 carpus length, superior distal margin with 1 palm seta, inferior margin first 0.67 covered in setal mat; *dactylus* length 1.3 width, length 0.36 propodus length, inferior margin distal 0.75 covered with scales, distal margin with 3 long, simple setae (Figures 3B, 27A). *Pereopod 7* (Figures 3C; 27B, C) *basis* superior margin with palm setae absent, inferior proximal margin with setal patch absent, inferior distal angle with long, simple setae absent; *ischium* length 2.9 width, superior margin with 3 long, simple RS; *merus* lobate, merus length 1.8 width, merus length 0.66 ischium length, superior distal angle with 4 RS, inferior margin with setal mat, inferior distal angle with biserrate setae absent; *carpus* length 2.5 width, carpus length 1.3 merus length, inferior margin with setal mat, superior distal angle with a cluster of 5 long, biserrate setae, superior distal angle with a cluster of 3 long, simple, RS, distomesial margin with a cluster of 3 long, biserrate setae, inferior distal angle with a cluster of 5 long, biserrate setae, 1 long, simple RS; *propodus* weakly curved, length 4.0 width, length 1.3 carpus length, inferior margin proximal 0.33 with setal mat, superior distal angle with 2 long, simple setae, inferior margin with 2 long, simple setae, with palm setae absent; *dactylus* length 1.8 width, dactylus length 0.28 propodus length, margin with scales, distal margin with 3 simple setae (Figures 3C; 27B, C).

*Penial* process length 2.5 basal width (Figure 21B, C).

*Pleopod 1* peduncle length 0.48 width, with a cluster of 3 coupling hooks; endopod mesial margin heavily covered in fine, simple setae; exopod length 1.7 width, ventral surface without fine, simple setae (Figure 4A). *Pleopod 2 appendix masculina* proximally swollen, distally narrowing, distal end curving mesially, straightening at distal tip, length 15.4 basal width (Figure 4B). *Pleopod 3* peduncle with a cluster of 3 coupling hooks, distolateral angle with 1 large, plumose seta (Figure 4C). *Pleopod 4* peduncle length 0.48 width, distolateral angle with 1 large, plumose seta; endopod distal apex 1 large, plumose seta; exopod distal margin with 2 plumose setae (Figure 4D). *Pleopod 5* exopod proximolateral margin with palm setae absent; exopod with transverse suture entire, endopod with 1 scale patch; exopod with 3 scale patches (Figure 4E). *Uropod* exopod length 2.3 width; rolled proximolateral margin weakening moving distally; mesial margin without setae; endopod length 2.5 width, extends past exopod, mesial margin without setae (Figures 4F; 21A, C, D).

#### Description of female.

*Body* length 2.7 width; pereonites 1–7 without tubercles, pereonite 7 distomesial margin convex (Figure 21E, F). *Pleon* with 1 posterior tubercle on either side of longitudinal axis (Figure 21E, F). *Pleotelson* length 2.6 width, dorsal surface with 2 medium tubercles on either side of longitudinal axis; posterior margin of pleotelson acuminate (Figure 21E, F). *Uropod* exopod proximolateral margin weakly rolled; endopod posterior margin tapering to evenly rounded tip, length 4.8 width, extends past exopod (Figure 21E, F).

**Size.** Largest ♂ to 8.4 mm, largest ♀ to 7.5 mm.

**Color.** Without chromatophores: preserved specimen pale buff, whitish.

#### Remarks.

*Exosphaeroma amplicauda* is most morphologically similar to *Exosphaeroma russellhansoni* sp. n. but can be distinguished by: pereonites 5 and 6 with one weak median tubercle, and one weak lateral tubercle; pereonite 7 with weak median process and paired lateral tubercles. (Figures 1A, B; 21A, D). Appendix masculina distal end curving mesially, straightening at distal tip, length 15.4 basal width (Figure 4B).

*Exosphaeroma russellhansoni* sp. n. is characterized by: pereonite 5–6 each without ornamentation, pereonite 7 with weak median process (Figures 9A, B; 23A, D). Appendix masculina distal end curving mesially, apex weakly hooked mesially, length 11.4 basal width (Figure 12B). *Exosphaeroma amplicauda* is strongly sexually dimorphic; females lack dorsal tubercles on pereonites 1–7. Overall for all species in this ‘species flock’ the males have a larger pleotelson and uropods. Weak pereon tubercles are visible only with SEM and not necessarily evident with light microscopy. Tubercles visible with light microscopy are figured in the line drawings (compare Figures 1 and 21).

We searched all probable museum collections for Stimpson’s type specimens, but to no avail (see Acknowledgements). It is highly likely that the type specimens are lost. The original and subsequent description (Stimpson 1857, 1858) do not allow for definitive identification of the species. There are five morphologically similar species in the northeast Pacific. A neotype is here designated to stabilize the use of the name *Exosphaeroma amplicauda* (Stimpson, 1857) and conserve Stimpson’s concept for the species.

We borrowed the types of *Sphaeroma octonctum* Richardson, 1899 (USNM Cat. No. 22574); Richardson (1899) noted that there were five specimens from the type locality, Monterey Bay). We received only four specimens–three had been previously dissected, some with pleopods removed, and only one specimen was entire. None of these specimens are adult males, and these specimens are indistinguishable from female *Exosphaeroma amplicauda* from Tomales Bay. We place *Sphaeroma octonctum* into junior synonymy with *Exosphaeroma amplicauda*.

#### Distribution.

California: Marin, Sonoma, and San Mateo Counties.

### Exosphaeroma paydenae sp. n.

Taxon classificationAnimaliaIsopodaSphaeromatidae

http://zoobank.org/D9B0B1E1-3BA3-4564-9AE9-F502034B553C

**Figure 5.**
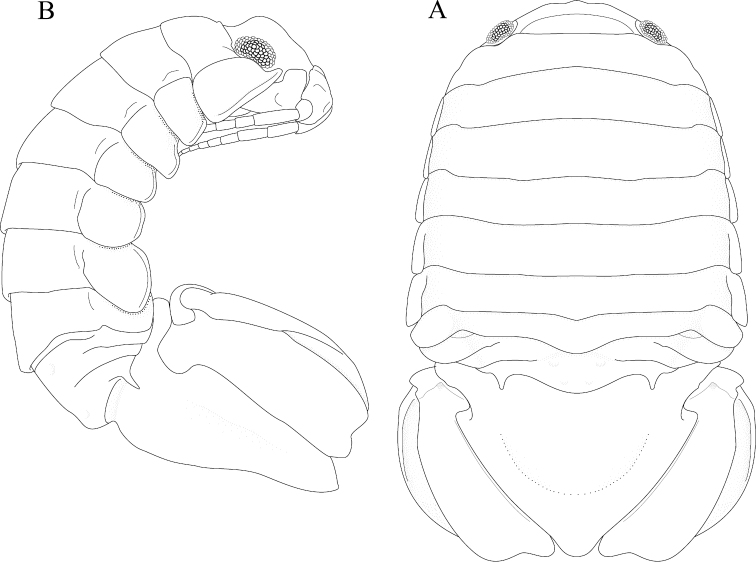
*Exosphaeroma paydenae* sp. n., male holotype USNM 20474. **A** dorsal **B** lateral.

**Figure 6.**
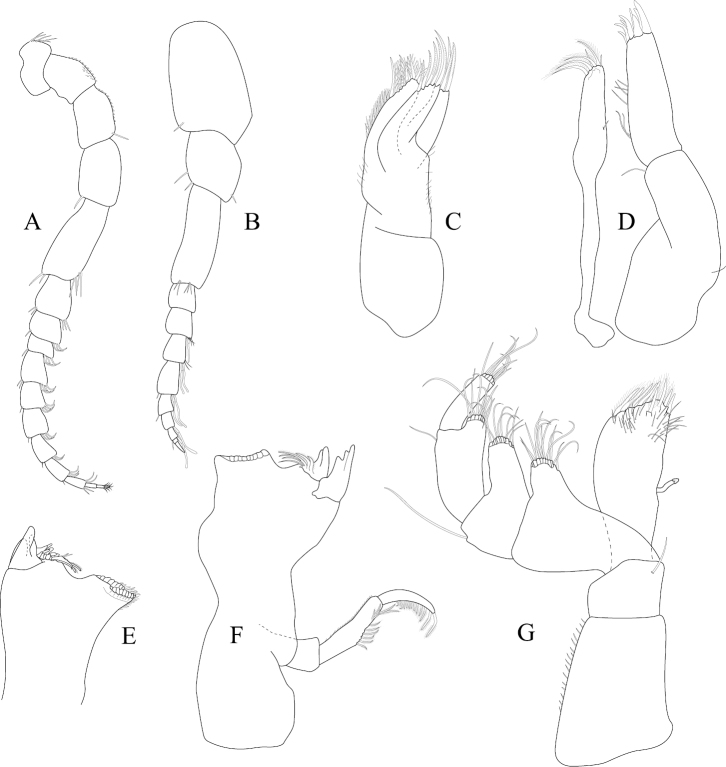
*Exosphaeroma paydenae* sp. n., male holotype USNM 20474. **A** right antenna **B** right antennula **C** right maxilla **D** right maxillula **E** right mandible **F** left mandible **G** right maxilliped.

**Figure 7.**
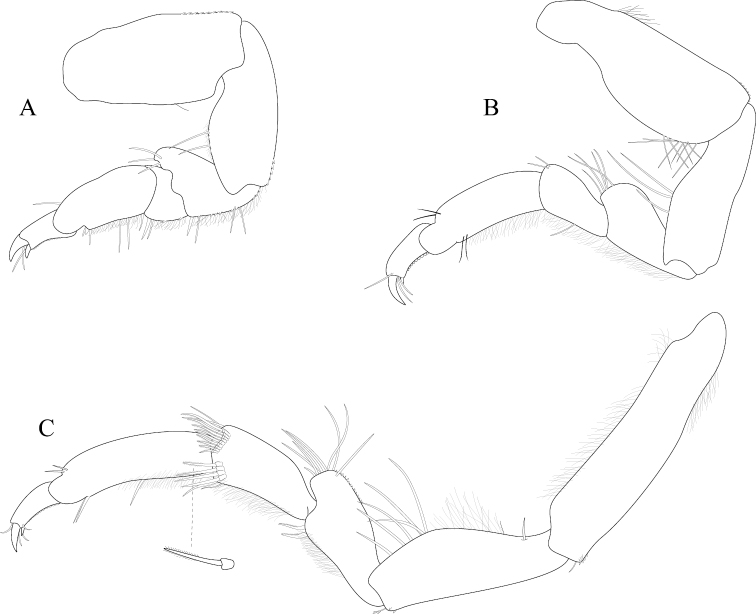
*Exosphaeroma paydenae* sp. n., male holotype USNM 20474. **A** right pereopod 1 **B** right pereopod 3 **C** right pereopod 7.

**Figure 8.**
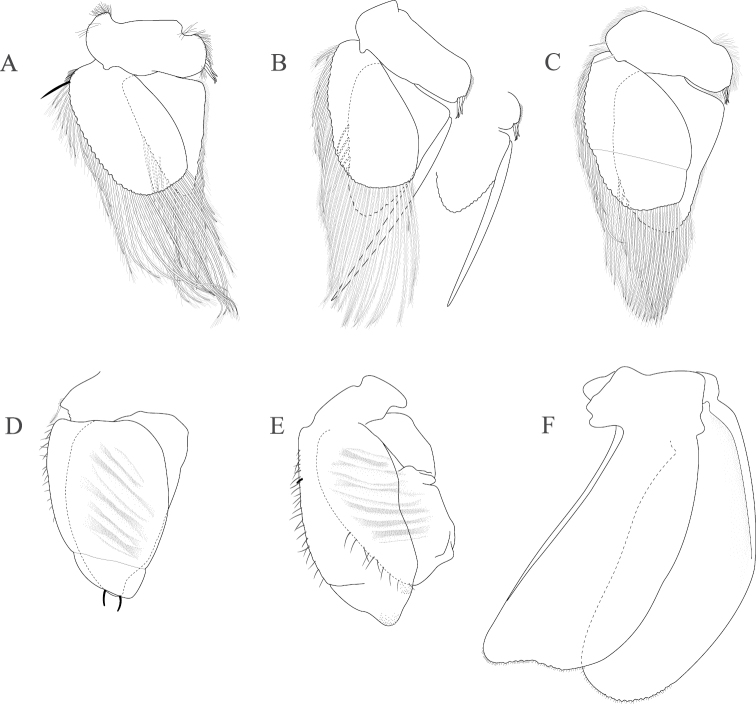
*Exosphaeroma paydenae* sp. n., male holotype USNM 20474. **A–E** right pleopods 1–5, respectively **F** right uropod.

Figures 5 , 6 , 7 , 8 , 22 , 28

Exosphaeroma amplicauda . – Gurjanova 1936: 122, fig. 69. – Kussakin 1979: 399, Figs 254, 255.

#### Material examined.

HOLOTYPE: ♂ (7.8 mm): Alaska, Aleutian Islands, Kiska Harbor, ~52.00°N, ~177.31°E, ca. 1873, beach, low water, USNM 20474, 211(1025), coll. W.H. Dall [Specimen label reads “Alaska, Kyoka Harbor.” per Marilyn Schotte, 15 Nov 2004 USNM 20474 reads “Aleutian Islands, Kiska Harbor” – maybe a transcription error on the label; specimens denoted as USNM 20474 are also possibly collected ca. 1873 similar to USNM 13312.] USNM 1251663.

PARATYPES: Allotype: ♀ (8.6 mm, whole animal figured): same locality as USNM 1251663, USNM 1251664. 1 ♂, 9 ♀, 2 juveniles, plus 1 ♂ and 1 ♀ prepared as SEM: all same locality as USNM 20474. 8 ♂ (8.0 mm), 1 ♂ broken: north coast of Amchitka, ~51.3°N, 179°E, 1 Jan 1873, USNM 13312, 284(1044), coll. W.H. Dall, USNM 1251665.

#### Description of male.

*Body* length 1.6 width; pereonites 5–7 each without ornamentation (Figures 5A, B; 22A, B). *Pleon* with 1 anterior weak tubercle on either side of longitudinal axis, 1 posterior weak tubercle on either side of longitudinal axis (Figures 5A, B; 22A). *Pleotelson* length 0.59 width, dorsal surface without ornamentation; ventrolateral ridge entire, with few setae (Figures 5A, B; 22A, D).

*Antennula* peduncle article 1 length 1.5 width, anterior medial margin with palm setae absent; article 2 length 1.1 width, inferior distal margin with palm setae absent; article 3 length 2.6 width; flagellum with 9 articles (Figure 6B). *Antenna* reaching posterior margin of pereonite 3, peduncle article 1 with fine, simple setae on superior margin; flagellum with 11 articles (Figure 6A).

*Left mandible* incisor with 3 cusps; lacinia mobilis with 2 cusps; lacinia mobilis spine row comprised of 6 curved, serrate spines (Figure 6F). *Right mandible* incisor with 3 cusps; spine row comprised of 7 curved, serrate spines; crushing surfaces strongly ridged (Figure 6E). *Maxillula* mesial lobe with 4 circumplumose RS, and 2 long, simple setae; lateral lobe with 7 long, curved, pectinate RS, gnathal surface with 1 curved, simple RS (Figure 6D). *Maxilla* mesial lobe with 1 long, straight RS, and 8 plumose RS on gnathal surface; middle lobe with 5 long, curved, pectinate RS; lateral lobe with 3 long, curved, pectinate RS (Figure 6C). *Maxilliped* endite distal surface with 8 plumose setae, and 1 simple RS; distomesial margin with 1 coupling hook; palp article 1 with 1 long, simple RS; article 2 distal apex with 12 long, simple RS; article 3 distal apex with 9 long, simple RS, lateral distal angle with 1 long, simple RS; article 4 distal apex with 9 long, simple RS, lateral distal angle with 1 long, simple RS; article 5 distal apex with 9 long, simple RS (Figure 6G).

*Pereopod 1* (Figure 7A) *basis* superior margin without palm setae, inferior distal angle without long, simple setae, inferior medial margin setal patch absent; *ischium* length 1.9 width, superior margin with 2 long, simple setae, inferior distal angle without long, simple setae; *merus* 0.58 ischium length, superior distal angle with 3 long, simple setae; *carpus* inferior distal angle with 2 long, simple setae; *propodus* length 2.1 width, 0.83 ischium length, superior distal angle with 1 long, simple seta, inferior margin with 3 long, simple setae; *dactylus* length 1.1 width, length 0.3 propodus length, inferior margin covered with fine scales, distal margin with 2 simple setae (Figure 7A). *Pereopod 3* (Figure 7B) *basis* superior margin without palm setae, inferior proximal margin with setal patch present; *ischium* length 2.6 width, superior margin with 3 long, simple RS, inferior distal angle with long, simple RS absent, and with setal patch absent; *merus* weakly lobate, length 1.4 width, 0.53 ischium length, superior distal angle with a cluster of 5 long, simple RS, inferior margin covered in setal mat; *carpus* length 0.88 merus length, 1.5 width, superior margin with 2 long, simple setae on distal angle, inferior margin with setal mat, and 1 long, simple seta; *propodus* weakly curved, length 2.9 width, 1.7 carpus length, superior distal margin without palm setae, inferior margin covered in setal mat; *dactylus* length 1.6 width, length 0.33 propodus length, inferior margin first 0.75 covered with scales, distal margin with 3 long, simple setae (Figure 7B). *Pereopod 7* (Figure 7C) *basis* superior margin with palm setae absent, inferior proximal margin with setal patch, inferior distal angle with long, simple setae absent; *ischium* length 2.6 width, superior margin with 7 long, simple RS; *merus* lobate, merus length 1.6 width, merus length 0.56 ischium length, superior distal angle with 9 RS, inferior margin with setal mat, inferior distal angle with 2 biserrate setae; *carpus* length 1.8 width, carpus length 0.96 merus length, inferior margin with setal mat, superior distal angle with a cluster of 9 long, biserrate setae, superior distal angle with a cluster of 2 long, simple, RS, inferior distal angle with a cluster of 4 long, biserrate setae, inferior distal angle with 1 long, simple RS; *propodus* weakly curved, length 3.9 width, length 1.8 carpus length, inferior margin first 0.75 with setal mat, inferior distal margin with 2 long, simple setae, and with palm setae absent; *dactylus* length 2.0 width, dactylus length 0.26 propodus length, inferior margin with fine scales, distal margin with 4 simple setae (Figure 7C).

*Penial* process length 3.2 basal width (Figure 22C, D).

*Pleopod 1* peduncle length 0.46 width, with a cluster of 3 coupling hooks; endopod mesial margin entirely covered with fine, simple setae; exopod length 1.7 width, ventral surface without fine, simple setae (Figure 8A). *Pleopod 2 appendix masculina* straight, distally narrowing, distal apex acute, length 16.0 basal width (Figure 8B). *Pleopod 3* peduncle with a cluster of 3 coupling hooks, distolateral angle with 2 long, simple setae (Figure 8C). *Pleopod 4* peduncle distolateral angle with 1 long, palm seta; endopod distal apex without plumose setae; exopod distal margin with 2 simple setae (Figure 8D). *Pleopod 5* exopod proximolateral margin with palm setae absent; exopod with transverse suture starting laterally moving mesially, incomplete; exopod with 2 scale patches (Figure 8E). *Uropod* exopod length 2.5 width; rolled proximolateral margin weakening moving toward lateral, medial margin; mesial margin without setae; endopod length 2.8 width, extends past exopod, mesial margin without setae (Figures 8F; 22A, B, D).

#### Description of female.

*Body* length 2.2 width; pereonites 1–7 without tubercles, pereonite 7 distomesial margin weakly convex (Figure 22E, F). *Pleon* with 1 posterior weak tubercle on either side of longitudinal axis (Figure 22E, F). *Pleotelson* length 1.8 width, dorsal surface with 2 tubercles on either side of longitudinal axis; posterior margin of pleotelson acuminate (Figure 22E, F). *Uropod* exopod proximolateral margin rolled weakly; endopod posterior margin tapering to an evenly rounded tip, length 2.9 width, extends past exopod (Figure 22E, F).

#### Size.

Largest ♂ 8.0 mm, largest ♀ 8.6 mm.

#### Color.

Without chromatophores. Preserved specimen pale cream.

#### Remarks.

*Exosphaeroma paydenae* sp. n., unlike other *Exosphaeroma* sp. in this ‘species flock’, lacks strong sexual dimorphism. Males have overall larger pleotelson and uropods than females. *Exosphaeroma paydenae* sp. n. is morphologically most similar to *Exosphaeroma russellhansoni* sp. n. *Exosphaeroma paydenae* sp. n. can be identified by: pereonites 1–7 without tubercles; pleon with one anterior weak tubercle on either side of longitudinal axis, one posterior weak tubercle on either side of longitudinal axis; pleotelson dorsal surface without ornamentation (Figures 5A, B; 22A, B).

**Figure 9.**
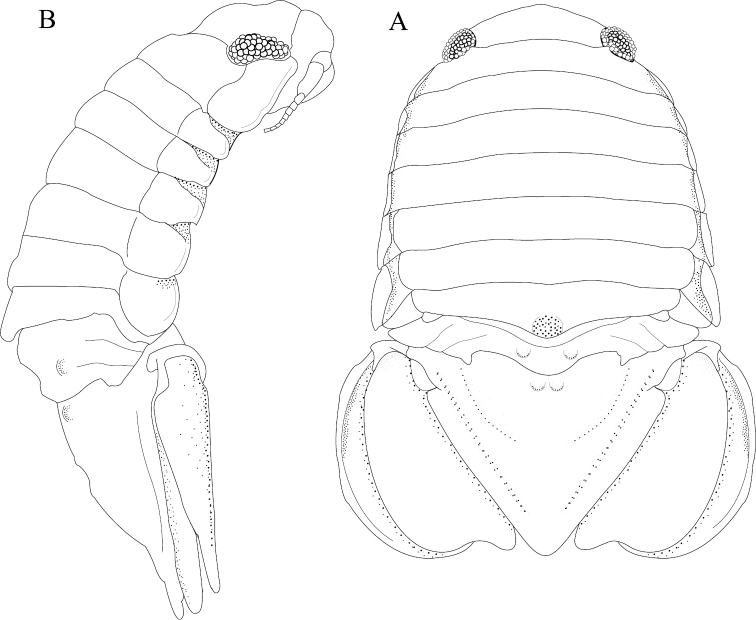
*Exosphaeroma russellhansoni* sp. n. male holotype LACM CR-2014.6. **A** dorsal **B** lateral.

*Exosphaeroma russellhansoni* sp. n., in contrast to *Exosphaeroma paydenae* has only one weak tubercle on either side of longitudinal axis of its pleon; pleotelson dorsum, with 2 small anterior tubercles (Figures 9A, B; 23A, D). Weak pereon tubercles are visible only with SEM and not necessarily evident with light microscopy. Tubercles visible with light microscopy are figured in the line drawings (compare Figures 5 and 22).

Kussakin (1979) provided new figures for what he considered to be specimens of *Exosphaeroma amplicauda* from Alaska. In his description he wrote “one sample (three specimens) from Alaska was examined from the collections of the Zoological Institute, Academy of Sciences of the USSR.” We here recognize the Alaska specimens as *Exosphaeroma paydenae* sp. n., which does not overlap in occurrence with species from further south, all described herein.

#### Distribution.

Alaska, Aleutians.

#### Etymology.

This species is named to honor LACM Trustee and long supporter of science at the Natural History Museum of Los Angeles County, Joan Payden. She is thanked for her gracious philanthropy which in part supported ARW as an undergraduate student researcher. ARW’s research experience describing and redescribing the *Exosphaeroma* along our coast piqued his interest in marine isopods and launched his career in Crustacea at the LACM.

### Exosphaeroma russellhansoni sp. n.

Taxon classificationAnimaliaIsopodaSphaeromatidae

http://zoobank.org/9A4E9501-0543-4615-B473-03F54F2C632A

**Figure 10.**
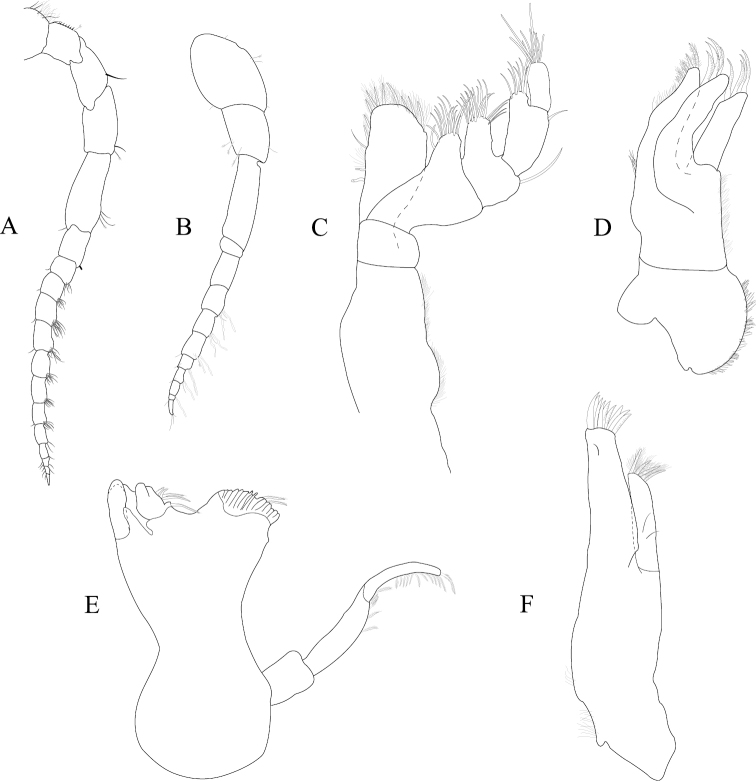
*Exosphaeroma russellhansoni* sp. n., male paratype LACM CR-2014.6. Male paratype RW04.010.3 **A** right antenna **B** right antennula, basal article broken **C** left maxilliped **D** left maxilla **E** male paratype left mandible **F** right maxillula.

**Figure 11.**
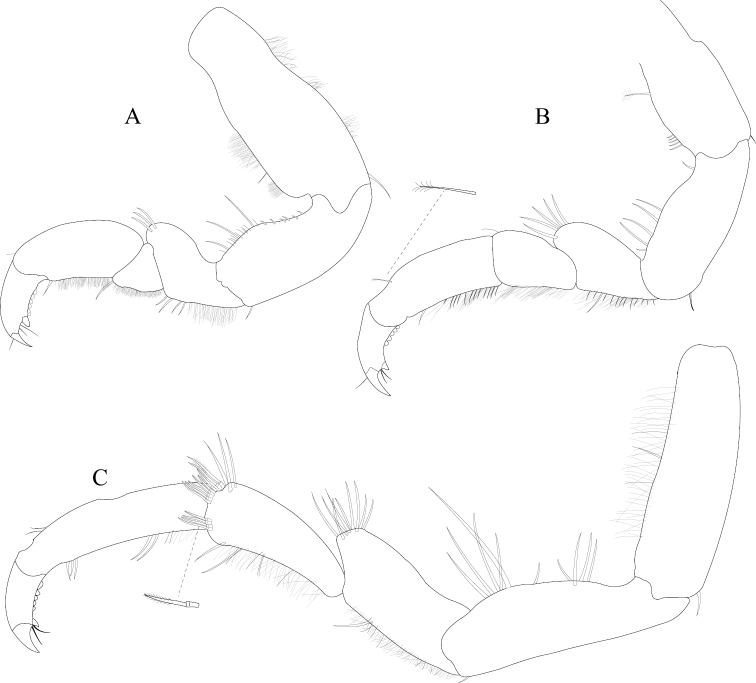
*Exosphaeroma russellhansoni* sp. n., male holotype LACM CR-2014.6. **A** left pereopod 1 **B** left pereopod 3 **C** left pereopod 7.

**Figure 12.**
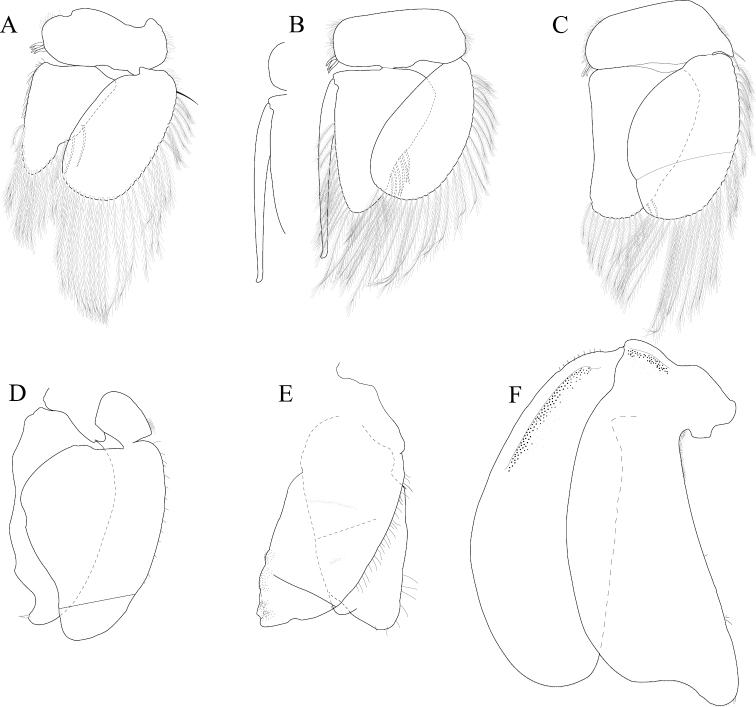
*Exosphaeroma russellhansoni* sp. n., male paratype LACM CR-2014.6. **A–E** left pleopods 1–5, respectively **F** left uropod.

Figures 9 , 10 , 11 , 12 , 23 , 28

#### Material examined.

HOLOTYPE: ♂ (7.0 mm): Washington, Puget Sound, Seattle, Puget Sound Naval Supply Depot, Smith Cove, ~47.5°N, ~122.2°W, under rocks in sand, 11 Aug 1973. A.0030, coll. E.W. Iverson. RW04.010.1, LACM CR-2014.6.

PARATYPES: Allotype gravid ♀ (6.7 mm): same data as holotype, LACM CR-2014.6. 1 ♂ dissected, appendages figured (RW04.010.3), 1 ♂ (RW04.010.4, LACM CR-2014.6.4) and 2 ♀ (6.7 mm RW04.010.5, LACM CR-2014.6.5) prepared as SEM, plus ~70 additional specimens (all life stages RW04.010.6): same locality as RW04.010, LACM CR-2014.6. 3 ♀ (7.2 mm), 1 subadult ♂: south end of San Juan Island, Cattle Point, 48.451°N, 122.967°W, rocky intertidal barnacles from *Semibalanus cariosus*, fixed and preserved in 95% ethanol, 7 Apr 2004, coll. R. Wetzer & N.D. Pentcheff. RW04.036.1, LACM CR-2014.7. 1 ♂ (7.9 mm), 1 subadult intermolt: northeast of San Juan Island, Reuben Tarte County Park, 48.612°N, 123.098°W, scrapings of vertical rock surface in intertidal, fixed and preserved in 95% ethanol, 9 Apr 2004, coll. R. Wetzer & N.D. Pentcheff. RW04.041.1, LACM CR-2014.8. 6 ♂ (8.3 mm), 4 ♀ (8.7 mm), 2 ♂ dissected for mandibles and figured: San Juan Island, old man’s farm, ~48.6°N, ~122.9°W, under rocks, 30 Jul 1950. USNM Acc. No. 187867, coll. L. Peternick and P. Illg. USNM 1251666. 3 ♂, 1 gravid ♀, 1 subadult: San Juan Islands, False Bay, 1 Aug 1975, transferred to 95% ethanol 5 Oct 2012, coll. R.R. Hessler. RW12.215.1, LACM CR-2014.9.

#### Description of male.

*Body* length 1.6 width; pereonite 5–6 each without ornamentation, pereonite 7 with weak median process (Figures 9A, B; 23A, D). *Pleon* with 1 medium tubercle on either side of longitudinal axis (Figures 9A, B; 23A, D). *Pleotelson* length 0.65 width, dorsal surface with 2 small anterior tubercles; ventrolateral ridge entire, with long setae (Figures 9A, B; 23A, C, D).

*Antennula* peduncle article 1 length 1.2 width, anterior medial margin with 1 palm seta; article 2 length 1.2 width, inferior distal margin with 3 palm setae; article 3 length 2.9 width; flagellum with 9 articles (Figure 10B). *Antenna* reaching medium margin of pereonite 2, peduncle article 1 with numerous fine simple setae on anterior posterior margin; flagellum with 13 articles (Figure 10A).

*Left mandible* incisor with 3 cusps; lacinia mobilis with 2 cusps; lacinia mobilis spine row comprised of 6 curved, serrate spines; crushing surfaces strongly ridged, with 2 serrate spines (Figure 10E). *Maxillula* mesial lobe with 4 circumplumose RS; lateral lobe with 9 long, curved, pectinate RS (Figure 10F). *Maxilla* mesial lobe with 6 plumose RS on gnathal surface; middle lobe with 4 long, curved, pectinate RS; lateral lobe with 4 long, curved, pectinate RS (Figure 10D). *Maxilliped* endite distal surface with 11 plumose setae; distomesial margin 2 coupling hooks, and 2 large stout plumose setae, and 1 large simple RS; palp article 2 distal apex with 15 long, simple RS; article 3 distal apex with 17 long, simple RS, lateral distal angle with 1 long, simple RS; article 4 distal apex with 8 long, simple RS, lateral distal angle with 1 long, simple RS; article 5 distal apex with 9 long, simple RS (Figure 10C).

*Pereopod 1* (Figure 11A) *basis* superior margin with 1 palm seta, inferior distal angle with 1 long, simple seta, inferior medial margin setal patch present; *ischium* length 1.9 width, superior margin with 3 long, simple setae, inferior distal angle with 1 long, simple seta; *merus* 0.45 ischium length, superior distal angle with 3 long, simple setae; *carpus* inferior distal angle with 1 long, simple seta; *propodus* length 2.3 width, 0.93 ischium length, superior distal angle without long, simple setae, inferior margin with 1 long, simple seta; *dactylus* length 1.5 width, length 0.46 propodus length, inferior margin covered with scales, distal margin with 3 simple setae (Figure 11A). *Pereopod 3* (Figure 11B) *basis* superior margin with 1 palm seta, inferior distal angle with 1 long simple seta, inferior proximal margin with setal patch absent; *ischium* length 2.4 width, superior margin with 5 long, simple RS, inferior distal angle with 1 simple RS, and with setal patch absent; *merus* lobate, length 0.95 width, 0.4 ischium length, superior distal angle with a cluster of 5 simple RS, inferior margin covered in setal mat; *carpus* length 1.3 merus length, 1.4 width, superior margin with 1 long, simple seta on distal angle, inferior margin with setal mat, and long, simple setae absent; *propodus* weakly curved, length 2.6 width, 1.7 carpus length, superior distal margin with 1 palm seta, inferior margin first 0.67 covered in setal mat; *dactylus* length 1.2 width, length 0.33 propodus length, inferior margin proximal half with scales, distal margin with 3 long, simple setae (Figure 11B). *Pereopod 7* (Figure 11C) *basis* superior margin with palm setae absent, inferior proximal margin with setal patch absent, inferior distal angle with 1 long, simple seta present; *ischium* length 3.0 width, superior margin with 10 long, simple RS; *merus* lobate, merus length 1.7 width, merus length 0.53 ischium length, superior distal angle with 9 RS, inferior margin with setal mat, inferior distal angle with biserrate setae absent; *carpus* length 2.5 width, carpus length 1.3 merus length, inferior margin with setal mat, superior distal angle with a cluster of 7 long, biserrate setae, superior distal angle with a cluster of 3 long, simple, RS, inferior distal angle with a cluster of 5 long, biserrate setae, inferior distal angle with 1 long, simple RS; *propodus* weakly curved, length 4.0 width, length 1.2 carpus length, inferior margin with setal mat absent, superior distal angle with 2 long, simple setae, inferior distal margin with 3 long, simple setae, and with palm setae absent; *dactylus* length 1.6 width, dactylus length 0.24 propodus length, inferior margin with scales, distal margin with 2 simple setae (Figure 11C).

*Penial* process length 2.7 basal width (Figure 23B).

*Pleopod 1* peduncle length 0.41 width, with a cluster of 4 coupling hooks; endopod mesial margin lightly covered in fine, simple setae; exopod length 1.8 width, ventral surface without fine, simple setae (Figure 12A). *Pleopod 2 appendix masculina* proximally swollen, distally narrowing, distal end curving mesially, apex weakly hooked mesially, length 11.4 basal width (Figure 12B). *Pleopod 3* peduncle with a cluster of 3 coupling hooks, distolateral angle with 2 long, simple setae (Figure 12C). *Pleopod 4* peduncle distolateral angle with fine setal patch; endopod distal apex 1 large, plumose seta; exopod distal margin without setae (Figure 12D). *Pleopod 5* exopod proximolateral margin with palm setae absent; exopod with distal transverse suture starting laterally, incomplete; exopod with 4 scale patches (Figure 12E). *Uropod* exopod length 2.4 width; rolled proximolateral margin weakening moving toward lateral, medial margin; mesial margin without setae; endopod length 2.7 width, extends past exopod, mesial proximal margin with setal patch (Figures 12F; 23A, C, D).

#### Description of female.

*Body* length 2.7 width; pereonites 1–7 without tubercles, pereonite 7 distomesial margin convex (Figure 23E, F). *Pleon* with 1 weak posterior tubercle on either side of longitudinal axis (Figure 23E, F). *Pleotelson* length 0.75 width, dorsum without tubercles; posterior margin of pleotelson acuminate (Figure 23E, F). *Uropod* exopod proximolateral margin rolled weakly; endopod length 3.7 width, extends past exopod (Figure 23E, F).

#### Size.

Largest ♂ 8.3 mm, largest ♀ 8.7 mm.

#### Color.

Without chromatophores. Preserved specimen pale buff, whitish.

#### Remarks.

*Exosphaeroma russellhansoni* sp. n. is morphologically most similar to *Exosphaeroma amplicauda* but can be easily distinguished by: pereonite 5–6 each without ornamentation, pereonite 7 with weak median process (Figures 9A, B; 23A, D). Appendix masculina distal end curving mesially, apex weakly hooked mesially, length 11.4 basal width (Figure 12B).

*Exosphaeroma amplicauda* is distinguished by: pereonites 5 and 6 with one weak median tubercle, and one weak lateral tubercle; pereonite 7 with weak median process and paired lateral tubercles (Figures 1A, B; 21A, D). Appendix masculina distal end curving mesially, straightening at distal tip, length 15.4 basal width (Figure 4B). *Exosphaeroma russellhansoni* sp. n. is strongly sexually dimorphic; females lacking dorsal tubercles on pereonites 1–7. Weak pereon tubercles are visible only with SEM, but not necessarily evident with light microscopy, and therefore are omitted from line drawings (compare Figures 9 and 23).

#### Distribution.

Washington, Puget Sound and San Juan Island.

#### Etymology.

Named to honor Russell Kenneth Hanson, ARW’s only maternal uncle who has shaped the person Adam is today by so graciously sharing with Adam his insatiable curiosity, life-long pursuit of perfection and tireless work ethic.

### Exosphaeroma pentcheffi sp. n.

Taxon classificationAnimaliaIsopodaSphaeromatidae

http://zoobank.org/82947847-B852-4628-AE6E-66DDCEEDCEDF

**Figure 13.**
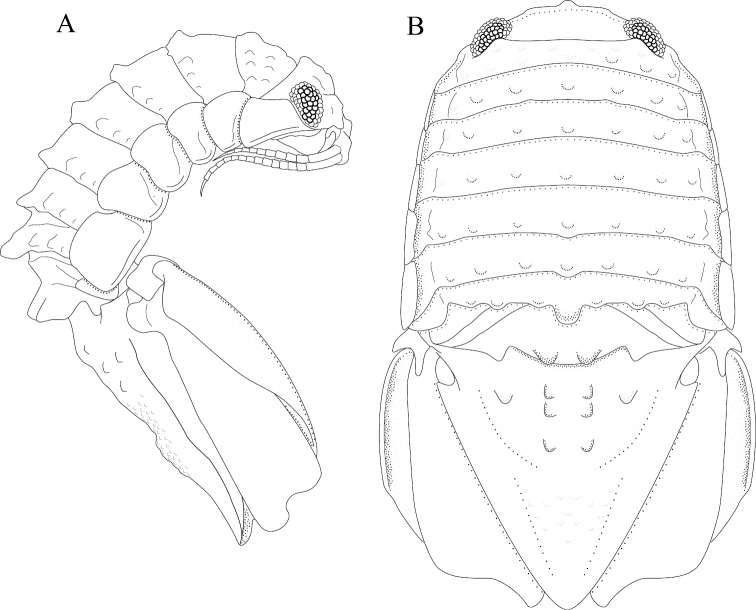
*Exosphaeroma pentcheffi* sp. n., male holotype LACM CR-2014.10. **A** lateral **B** dorsal.

**Figure 14.**
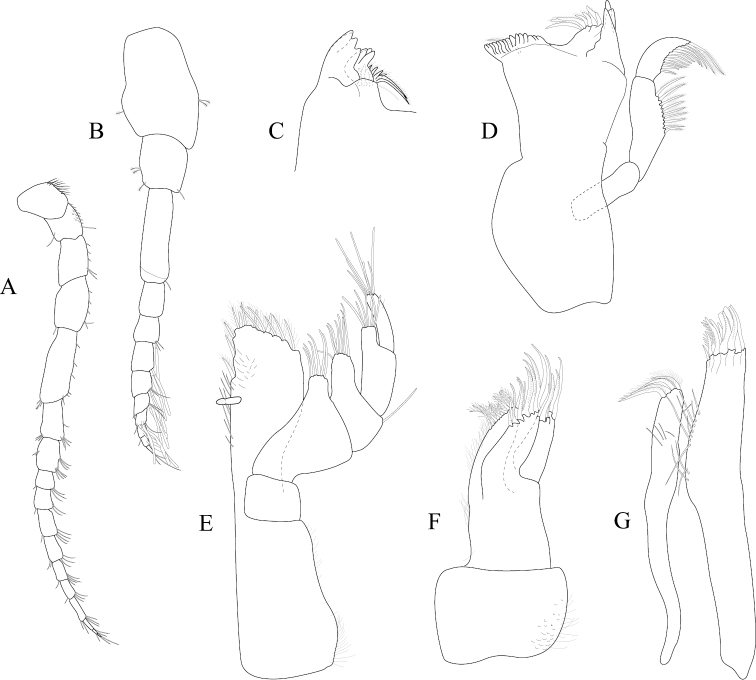
*Exosphaeroma pentcheffi* sp. n., male holotype LACM CR-2014.10. **A** left antenna **B** left antennula **C** left mandible **D** right mandible **E** left maxilliped **F** left maxilla **G** left maxillula.

**Figure 15.**
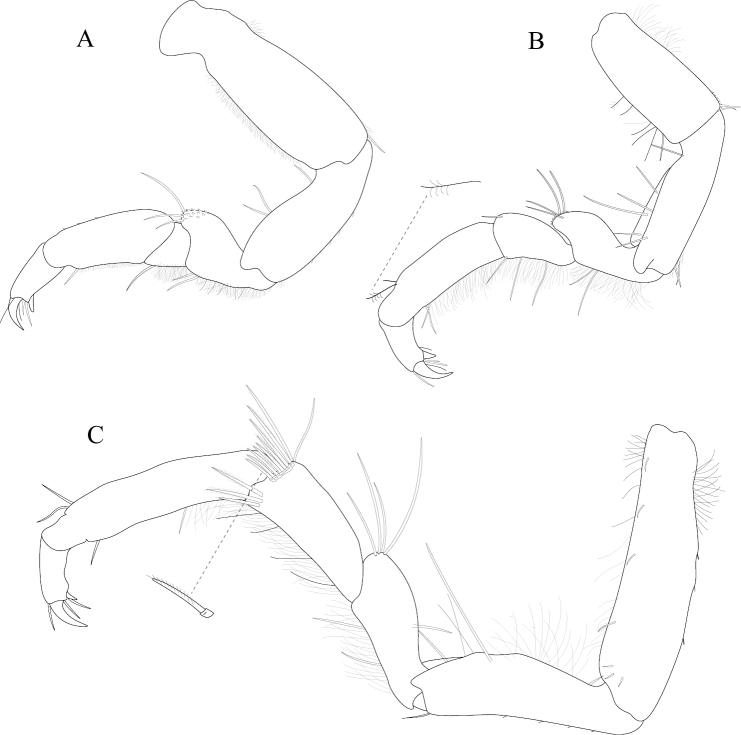
*Exosphaeroma pentcheffi* sp. n., male holotype LACM CR-2014.10. **A** left pereopod 1 **B** left pereopod 3 **C** left pereopod 7.

**Figure 16.**
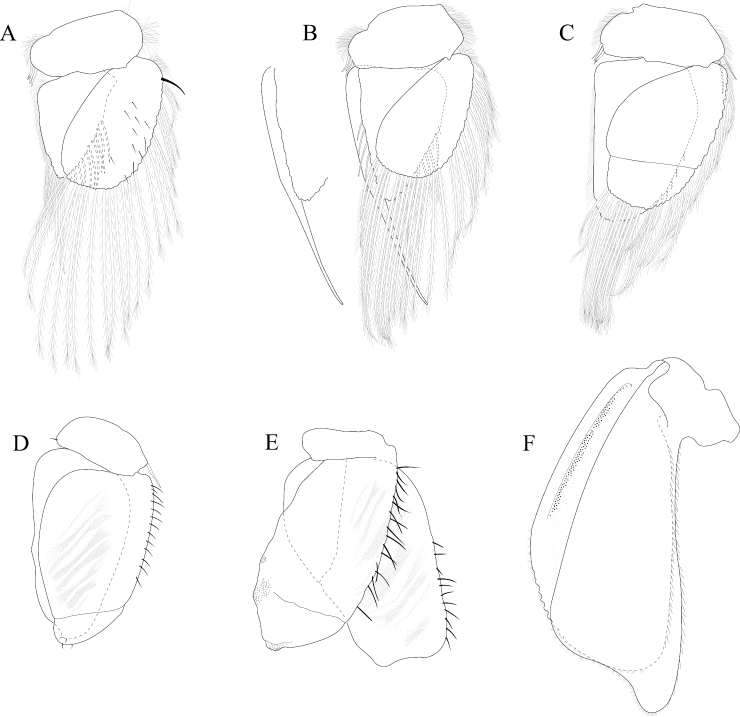
*Exosphaeroma pentcheffi* sp. n., male holotype LACM CR-2014.10. **A–E** left pleopods 1–5, respectively **F** left uropod.

Figures 13 , 14 , 15 , 16 , 24 , 28

#### Material examined.

HOLOTYPE ♂ (4.6 mm): California, Los Angeles County, Palos Verdes Peninsula, Pt. Fermin, shore at Paseo del Mar, ~0.5 mi. W of Gaffey Street, 33.71°N, 118.3°W, mid-low intertidal, chipping overhanging rock with hammer and *Phragmatopoma* tubes on underside of rock, 0.99 m depth, fixed and preserved in 95% ethanol, 27 Mar 2004, coll. R. Wetzer, N.D. Pentcheff, and LMU students. RW04.030.1, LACM CR-2014.10.

PARATYPES: Allotype ♀ (4.6 mm) (whole animal figured): shore at Paseo del Mar, ~0.5 mi. W of Gaffey Street, 33.71°N, 118.3°W, mostly barnacles, some algal turf, medium to high intertidal, paint scraper, fixed and preserved in 95% ethanol, 16 Feb 2004, coll. R. Wetzer. RW04.002.1, LACM CR-2014.11. 1 ♂ accidently destroyed after being imaged (RW04.255, LACM CR-2014.12), 1 ♂, 3 ♀ (RW04.030.2), plus 1 ♀ (4.6 mm) prepared as SEM: RW04.030.3, LACM CR-2014.10. 3♀(RW04.002.2), plus 1 ♂ (RW04.002.3) and 1 ♀ (RW04.002.4) prepared for SEM: shore at Paseo del Mar, ~0.5 mi. W of Gaffey Street, 33.71°N, 118.3°W, mostly barnacles, some algal turf, medium to high intertidal, paint scrapper, fixed and preserved in 95% ethanol, 16 Feb 2004, coll. R. Wetzer. RW04.002, LACM CR-2014.11. 1 ♂ (5.1 mm), 2 ♀: shore at Paseo del Mar, ~0.5 mi. W of Gaffey Street, 33.71°N, 118.3°W, found in bottom of bucket with sea stars, mid- to low intertidal, fixed and preserved in 95% ethanol, 16 Feb 2004. Loyola Marymount University Invertebrate Class, N.D. Pentcheff, coll. E. Pattison and K. Stanley. RW04.003.1, LACM CR-2014.13.

#### Description of male.

*Body* length 1.8 width; pereonites 5–6 each with 7 longitudinal rows of strong tubercles, pereonite 7 with strong median process with 3 lateral tubercles (Figures 13A, B; 24A, D). *Pleon* with 1 medium tubercle on posterior margin, on either side of longitudinal axis (Figures 13A, B; 24A, D). *Pleotelson* length 0.85 width, dorsal surface with 3 strong medial tubercles on either side of the longitudinal axis, with 1 strong medial tubercle between the longitudinal axis and lateral margin, pleotelson covered with numerous, additional, small tubercles; ventrolateral ridge extending posteriorly 0.80 of total length, with long setae (Figures 13A, B; 24A, C, D).

*Antennula* peduncle article 1 length 1.4 width, anterior medial margin with 2 palm setae; article 2 length 1.1 width, inferior distal margin with 3 palm setae; article 3 length 3.1 width; flagellum with 9 articles (Figure 14B). *Antenna* reaching anterior margin of pereonite 2, peduncle article 1 with fine, simple setae on superior margin; flagellum with 11 articles (Figure 14A).

*Left mandible* incisor with 4 cusps; lacinia mobilis with 3 cusps; lacinia mobilis spine row comprised of 8 curved, serrate spines, and 1 curved, robust, simple spine (Figure 14C). *Right mandible* incisor with 3 cusps; spine row comprised of 7 curved, serrate spines; crushing surfaces strongly ridged, with 1 serrate spine (Figure 14D). *Maxillula* mesial lobe with 4 circumplumose RS; lateral lobe with 10 long, curved, pectinate RS, gnathal surface with 1 curved, simple RS (Figure 14G). *Maxilla* mesial lobe with 1 long, straight RS, and 8 plumose RS on gnathal surface; middle lobe with 8 long, curved, pectinate RS; lateral lobe with 5 long, curved, pectinate RS (Figure 14F). *Maxilliped* endite distal surface with 7 plumose setae, and 3 simple RS; distomesial margin with 1 coupling hook, and 3 large stout plumose setae; palp article 2 distal apex with 6 long, simple RS; article 3 distal apex with 8 long, simple RS, lateral distal angle with 1 long, simple RS; article 4 distal apex with 7 long, simple RS; article 5 distal apex with 7 long, simple RS (Figure 14E).

*Pereopod 1* (Figure 15A) *basis* superior margin without palm setae, inferior distal angle with 1 long, simple seta, inferior medial margin setal patch absent; *ischium* length 2.3 width, superior margin with 3 long, simple setae, inferior distal angle without long, simple setae; *merus* 0.50 ischium length, superior distal angle with 3 long, simple setae; *carpus* inferior distal angle with 1 long, simple seta; *propodus* length 2.7 width, 1.0 ischium length, superior distal angle without long, simple setae, inferior margin with 1 long, simple seta; *dactylus* length 1.4 width, length 0.36 propodus length, inferior margin without setal scales, distal margin with 4 simple setae (Figure 15A). *Pereopod 3* (Figure 15B) *basis* superior margin with 1 palm seta, inferior distal angle with 2 long simple setae, inferior proximal margin with setal patch present; *ischium* length 3.1 width, superior margin with 6 long, simple RS, inferior distal angle with 2 long, simple RS, and with setal patch present; *merus* lobate, length 2.2 width, 0.73 ischium length, superior distal angle with 4 long, simple RS, inferior margin covered in setal mat; *carpus* superior margin with 1 long, simple seta on distal angle, inferior margin with setal mat, and 3 long, simple setae; *propodus* weakly curved, length 3.2 width, 1.8 carpus length, superior distal margin with 1 palm seta, inferior margin covered in setal mat; *dactylus* length 1.6 width, length 0.30 propodus length, inferior margin without scales, distal margin with 4 long, simple setae (Figure 15B). *Pereopod 7* (Figure 15C) *basis* superior margin with 1 palm seta, inferior proximal margin with setal patch, inferior distal angle with long, simple setae absent; *ischium* length 2.8 width, superior margin with 2 long, simple RS; *merus* lobate, merus length 2.1 width, merus length 0.63 ischium length, superior distal angle with 4 RS, inferior margin with setal mat, inferior distal angle with biserrate setae absent; *carpus* length 2.3 width, carpus length 1.1 merus length, inferior margin with setal mat, superior distal angle with a cluster of 8 long, biserrate setae, superior distal angle with a cluster of 2 long, simple, RS, inferior distal angle with a cluster of 3 long, biserrate setae; *propodus* weakly, curved, length 5.0 width, length 1.6 carpus length, inferior margin first 0.33 with setal mat, superior distal angle with 2 long, simple setae, inferior margin with 2 long, simple setae, and with palm setae absent; *dactylus* length 1.7 width, dactylus length 0.22 propodus length, inferior margin without fine scales, distal margin with 3 long, simple setae (Figure 15C).

*Penial* process length 3.0 basal width (Figure 24B).

*Pleopod 1* peduncle length 0.56 width, with a cluster of 3 coupling hooks; endopod mesial margin covered in fine, simple setae; exopod length 1.7 width, ventral surface with fine, simple setae (Figure 16A). *Pleopod 2 appendix masculina* distally narrowing to an acute rounded tip, length 15 basal width (Figure 16B). *Pleopod 3* peduncle with a cluster of 3 coupling hooks, distolateral angle with 2 large, simple setae (Figure 16C). *Pleopod 4* peduncle length 0.46 width, distolateral angle with 1 large, simple seta; endopod distal apex without plumose setae; exopod distal margin with 2 simple setae (Figure 16D). *Pleopod 5* exopod proximolateral margin with palm setae absent; exopod with transverse suture starting laterally moving mesially, incomplete; exopod with 4 scale patches (Figure 16E). *Uropod* exopod length 2.4 width; rolled proximolateral margin weakening moving toward lateral, distal margin; mesial margin with evenly spaced fine simple setae; endopod length 2.8 width, extends past exopod, distal apex with short, simple setal patch, dorsal surface covered with numerous small tubercles, mesial margin with evenly spaced fine simple setae (Figures 16F; 24A, C, D).

#### Description of female.

*Body* length 2.3 width; pereonites 2–6 each with 7 longitudinal rows of strong tubercles, pereonite 7 distomesial margin convex with strong median process, and 3 lateral tubercles (Figure 24E, F). *Pleon* with 1 posterior strong tubercle on either side of longitudinal axis (Figure 24E, F). *Pleotelson* length 0.61 width, dorsal surface with 3 strong medial tubercles on either side of the longitudinal axis, with 1 strong medial tubercle between the longitudinal axis and lateral margin, pleotelson covered with numerous, additional, small tubercles (Figure 24E, F). *Uropod* exopod proximolateral margin rolled; endopod length 3.6 width, extends past exopod, dorsal surface covered with numerous small tubercles, mesial margin without setae (Figure 24E, F).

#### Size.

Largest ♂ 6.8 mm, largest ♀ 4.6 mm.

#### Colour.

No chromatophores: preserved specimen pale buff, whitish.

#### Remarks.

*Exosphaeroma pentcheffi* sp. n. unlike the other *Exosphaeroma* species in this ‘species flock’ lacks strong sexual dimorphism and is unique in that females shares the same dorsal ornamentation as males; males differ from females in having slightly stronger tubercles, longer pleotelson and longer uropods. Females of *Exosphaeroma pentcheffi* sp. n. are the only females of this ‘species flock’ that can reliably be identified at the species level. *Exosphaeroma pentcheffi* sp. n. males can be identified by: pereonites 5 and 6 having 7 longitudinal rows of strong tubercles, pereonite 7 with a strong median process with 3 lateral tubercles; pleotelson dorsum with 3 strong medial tubercles on either side of the longitudinal axis, with 1 strong medial tubercle between the longitudinal axis and lateral margin, pleotelson covered with numerous, additional, small tubercles (Figures 13A, B; 24A, D, E, F). Weak pereon tubercles are visible only with SEM and not necessarily evident with light microscopy. Tubercles visible with light microscopy are figured in the line drawings (compare Figures 13 and 24).

#### Distribution.

California, Los Angeles County, Palos Verdes Peninsula.

#### Etymology.

This beautiful species is named for N. Dean Pentcheff, expert isopod collector, superb field and dive buddy, travel companion and IT support par excellence. Dean is commended for his reliable patience, support and solid friendship.

### Exosphaeroma aphrodita

Taxon classificationAnimaliaIsopodaSphaeromatidae

Boone, 1923

**Figure 17.**
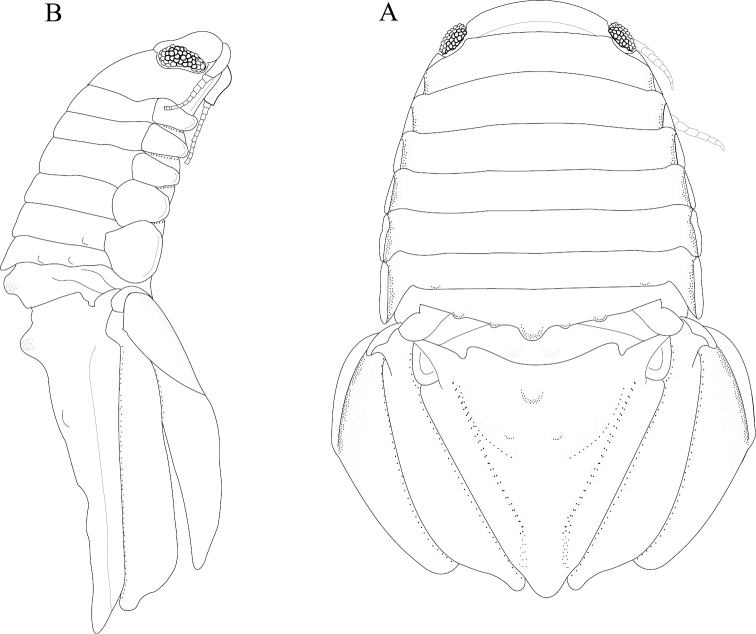
*Exosphaeroma aphrodita* male lectotype LACM CR-2014.14. **A** dorsal **B** lateral.

**Figure 18.**
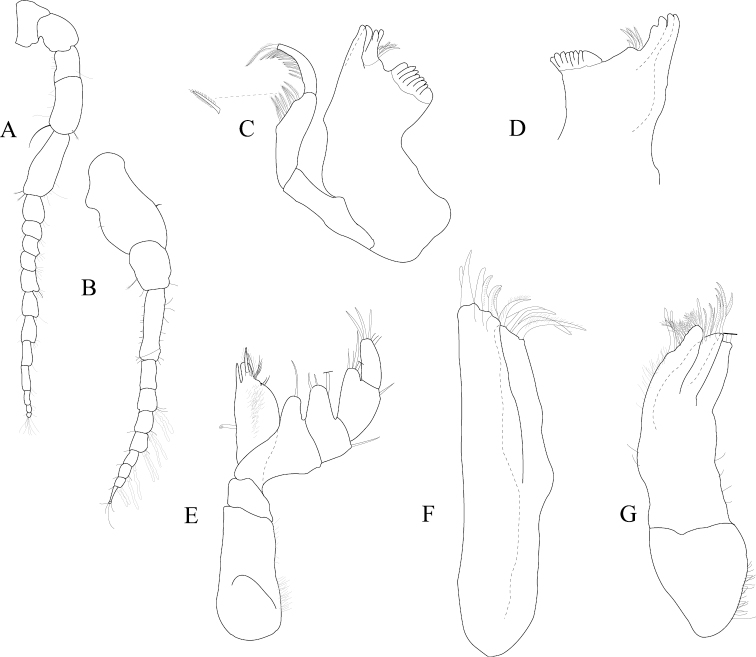
*Exosphaeroma aphrodita* male lectotype LACM CR-2014.14. **A** left anntenna **B** left antennula **C** left mandible **D** right mandible **E** left maxilliped **F** left maxillula **G** left maxilla.

**Figure 19.**
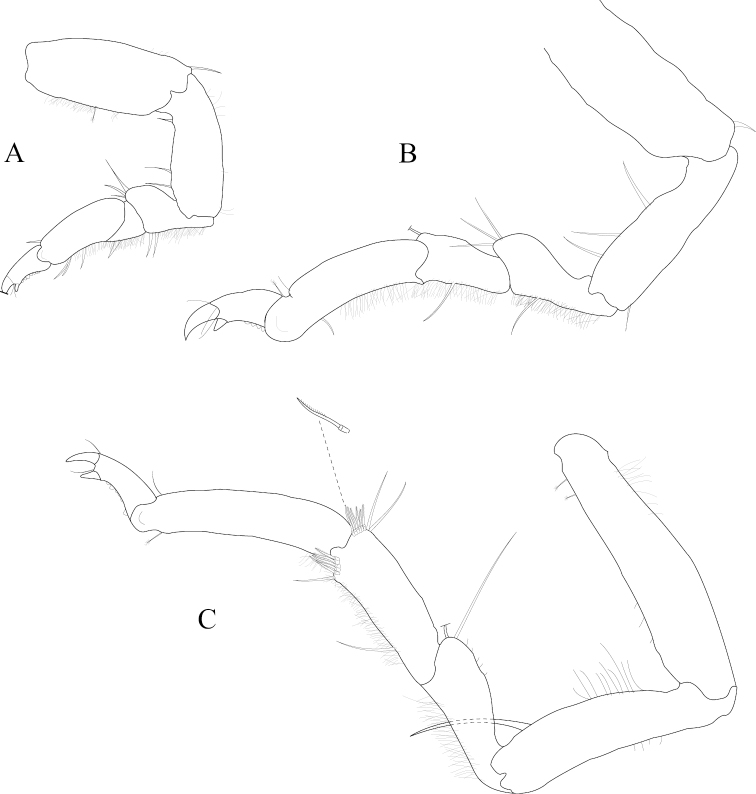
*Exosphaeroma aphrodita* male lectotype LACM CR-2014.14. **A** left pereopod 1 **B** left pereopod 3 **C** left pereopod 7.

**Figure 20.**
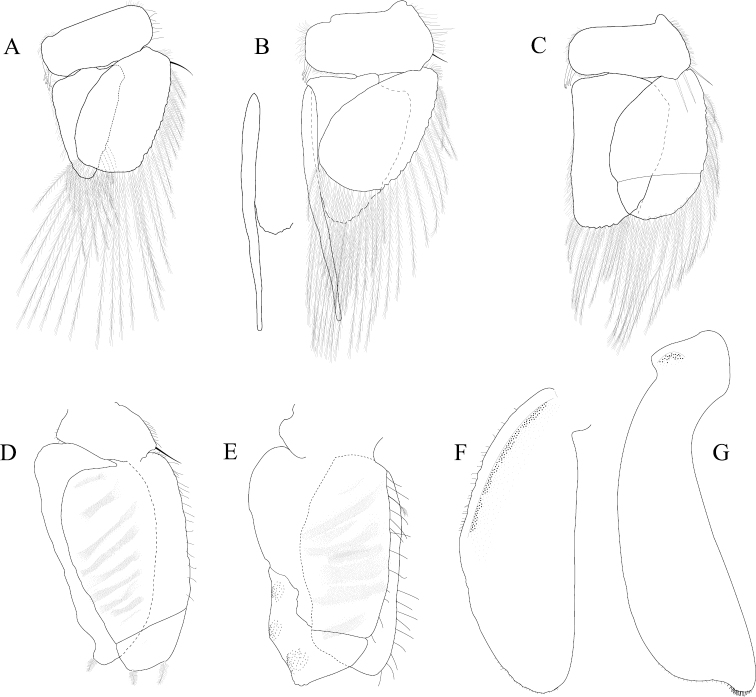
*Exosphaeroma aphrodita* male lectotype LACM CR-2014.14. **A–E** left pleopods 1–5, respectively **F** left uropod exopod **G** left uropod endopod.

Figures 17 , 18 , 19 , 20 , 25 , 28

Exosphaeroma aphrodita Boone, 1923. – Bruce 2003: 369. – Espinosa and Hendrickx 2006: 238. – Brusca et al. 2007: 537. – Bruce and Schotte 2012: online.

#### Material examined.

LECTOTYPE, here designated: 1 ♂ USNM 1251667 with mandibles dissected: California, San Diego County, La Jolla, Scripps Institute pier pilings, 6 Nov 1915. USNM Acc. No. 53848, #1045-1-4. Identified as *Exosphaeroma amplicauda* by P.L. Boone.

PARALECTOTYPES: 5 ♂ (USNM 1251667), with mandibles dissected. USNM 53848.

Non-type Material: 1 ♂: Scripps, ~32.87°N, ~117.26°W, littoral in algae, March 1938, coll. Olga Hartman and Loyola e Silva. USNM 1251668. 1 ♂ (RW01.002.1), 1 ♂, 3 ♀, (RW01.002.2) plus 1 ♂ (RW01.002.3) and 3 ♀ (RW01.002.4) prepared as SEM: Scripps Institute of Oceanography, beneath seaward end of Scripps Pier, ~32.87°N, ~117.26°W, to 8 m, among detritus at base of pilings, water temp. 59 °F, SCUBA, fixed and preserved in 95%, 7 Jan 2001, coll. T. Haney. RW01.002, LACM CR-2014.14. 1 ♂ (broken): San Diego, pilings, 1 Jul 1996. USNM Acc. No. 180084, Sta. No. 256. USNM 1251669.

#### Description of male.

*Body* length 2.0 width; pereonites 5 without ornamentation, pereonite 6 with 1 lateral weak tubercle, pereonite 7 with weak median process, and paired weak lateral tubercles (Figures 17A, B; 25A, C). *Pleon* with 1 medium tubercle on either side of longitudinal axis (Figures 17A, B; 25A, C). *Pleotelson* length 0.89 width, dorsal surface with 1 anterior median strong tubercle and 2 weak medial tubercles; ventrolateral ridge extending posteriorly 0.75 of total length, with long setae (Figures 17A, B; 25A, B, C).

*Antennula* peduncle article 1 length 1.5 width, anterior medial margin with palm setae absent; article 2 length 1.3 width, inferior distal margin with 1 palm seta; article 3 length 2.9 width; flagellum with 8 articles (Figure 18B). *Antenna* reaching posterior margin of pereonite 3, peduncle article 1 superior margin without palm setae; flagellum with 12 articles (Figure 18A).

*Left mandible* incisor with 2 cusps; lacinia mobilis with 2 cusps; lacinia mobilis spine row comprised of 6 curved, serrate spines (Figure 18C). *Right mandible* incisor with 4 cusps; spine row comprised of 5 curved, serrate spines; crushing surfaces strongly ridged (Figure 18D). *Maxillula* mesial lobe with 5 circumplumose RS; lateral lobe with 6 long, curved, pectinate RS (Figure 18F). *Maxilla* mesial lobe with 3 long, curved RS, and 6 plumose RS on gnathal surface; middle lobe with 8 long, curved, pectinate RS; and 7 long, curved RS; lateral lobe with 3 long, curved, pectinate RS (Figure 18G). *Maxilliped* endite distal surface with 2 plumose setae; distomesial margin with 1 coupling hook; palp article 2 distal apex with 1 long, simple RS; article 3 distal apex with 3 long, simple RS, lateral distal angle with 1 long, simple RS; article 4 distal apex with 3 long, simple RS, lateral distal angle with 1 long, simple RS; article 5 distal apex with 3 long, simple RS (Figure 18E).

*Pereopod 1* (Figure 19A) *basis* superior margin with 1 palm seta, inferior distal angle with 1 long, simple seta, inferior medial margin setal patch absent; *ischium* length 2.3 width, superior margin with 3 long, simple setae, inferior distal angle without long, simple setae; *merus* 0.40 ischium length, superior distal angle with 3 long, simple setae; *carpus* inferior distal angle with 1 long, simple seta; *propodus* length 2.1 width, 0.60 ischium length, superior distal angle with 1 long, simple seta, inferior margin with 3 long, simple setae; *dactylus* length 1.5 width, length 0.55 propodus length, inferior margin distal 0.67 covered with scales, distal margin with 2 simple setae (Figure 19A). *Pereopod 3* (Figure 19B) *basis* superior margin without palm setae, inferior distal angle with 1 long simple seta, inferior proximal margin with setal patch absent; *ischium* length 3.2 width, superior margin with 3 long, simple RS, inferior distal angle with 1 simple RS, and with setal patch absent; *merus* lobate, length 1.4 width, 0.56 ischium length, superior distal angle with a cluster of 3 RS, inferior margin covered in setal mat; *carpus* length 0.90 merus length, 1.9 width, superior margin with 1 long, simple seta on distal angle, inferior margin with setal mat, and 1 long, simple seta; *propodus* weakly curved, length 3.2 width, 2.0 carpus length, superior distal margin with 1 palm seta, inferior margin first 0.67 covered in setal mat; *dactylus* length 1.3 width, length 0.45 propodus length, inferior margin with scales, distal margin with 3 long, simple setae (Figure 19B). *Pereopod 7* (Figure 19C) *basis* superior margin with 2 palm setae, inferior proximal margin with setal patch, inferior distal angle with long, simple setae absent; *ischium* length 3.7 width, superior margin with 1 long, simple RS; *merus* weakly lobate, merus length 2.0 width, merus length 0.65 ischium length, superior distal angle with 2 RS, inferior margin with setal mat, inferior distal angle with biserrate setae absent; *carpus* length 2.9 width, carpus length 1.1 merus length, inferior margin with setal mat, superior distal angle with a cluster of 6 long, biserrate setae, superior distal angle with a cluster of 2 long, simple, RS, inferior distal angle with a cluster of 5 long, biserrate setae, inferior distal angle with 1 long, simple RS; *propodus* weakly curved, length 4.2 width, length 1.3 carpus length, inferior margin with setal patch, superior distal angle with 1 long, simple seta, inferior distal margin with simple, setae absent, and with 1 palm seta; *dactylus* length 2.0 width, dactylus length 0.27 propodus length, inferior margin with scales starting medially moving distally, distal margin with 2 simple setae (Figure 19C).

*Penial* process length 3.1 basal width (Figure 25B, D).

*Pleopod 1* peduncle length 0.42 width, with a cluster of 4 coupling hooks; endopod mesial margin lightly covered in fine, simple setae; exopod length 1.6 width, ventral surface without fine, simple setae (Figure 20A). *Pleopod 2 appendix masculina* apically narrowly rounded, length 13.0 basal width (Figure 20B). *Pleopod 3* peduncle with a cluster of 3 coupling hooks, distolateral angle with 3 large, simple setae (Figure 20C). *Pleopod 4* peduncle length 0.61 width, distolateral angle with 1 large, simple seta; endopod distal apex 1 large, plumose seta; exopod distal margin with 2 plumose setae (Figure 20D). *Pleopod 5* exopod proximolateral margin with 1 palm seta; exopod with transverse suture entire; exopod with 3 scale patches (Figure 20E). *Uropod* exopod length 2.4 width; rolled proximolateral margin weakening moving toward lateral, medial margin; mesial margin without setae; endopod length 3.1 width, extends past exopod, mesial margin without setae (Figures 20F, G; 25A, B, C).

#### Description of female.

*Body* length 2.9 width; pereonites 1–7 without tubercles, pereonite 7 distomesial margin weakly convex (Figure 25E, F). *Pleon* with 1 weak posterior tubercle on either side of longitudinal axis (Figure 25E, F). *Pleotelson* length 0.65 width, dorsal surface without visible tubercles; posterior margin of pleotelson acuminate (Figure 25E, F).

*Uropod* exopod rolled proximolateral margin rolled weakly; endopod length 3.4 width, extends past exopod, mesial margin without setae (Figure 25E, F).

#### Size.

Largest ♂ 8.3 mm, largest ♀ 8.7 mm.

#### Color.

No chromatophores: preserved specimen pale buff, whitish.

#### Remarks.

*Exosphaeroma aphrodita* can best be identified by: pereonite 5 without ornamentation, pereonite 6 with one lateral weak tubercle, pereonite 7 with weak median process, and paired weak lateral tubercles; pleotelson dorsum with one anterior median strong tubercle and two weak medial tubercles. *Exosphaeroma aphrodita* is strongly sexually dimorphic; females lack dorsal tubercles on the pereonites. Weak pereon tubercles are visible only with SEM and not necessarily evident with light microscopy. Tubercles visible with light microscopy are figured in the line drawings (compare Figures 17 and 25).

*Exosphaeroma aphrodita*, considered *nomen dubium* by Brusca et al. (2007), is here revalidated. Pearl Lee Boone described this species and several other isopods and tanaids without providing figures (Boone 1923, pp. 147–156). The original description states that “the type and additional material were collected at La Jolla, California and are in the collections of the United States National Museum.” We examined all of the USNM material available. We conclude that the species is valid.

#### Distribution.

California, San Diego–La Jolla.

**Figure 21.**
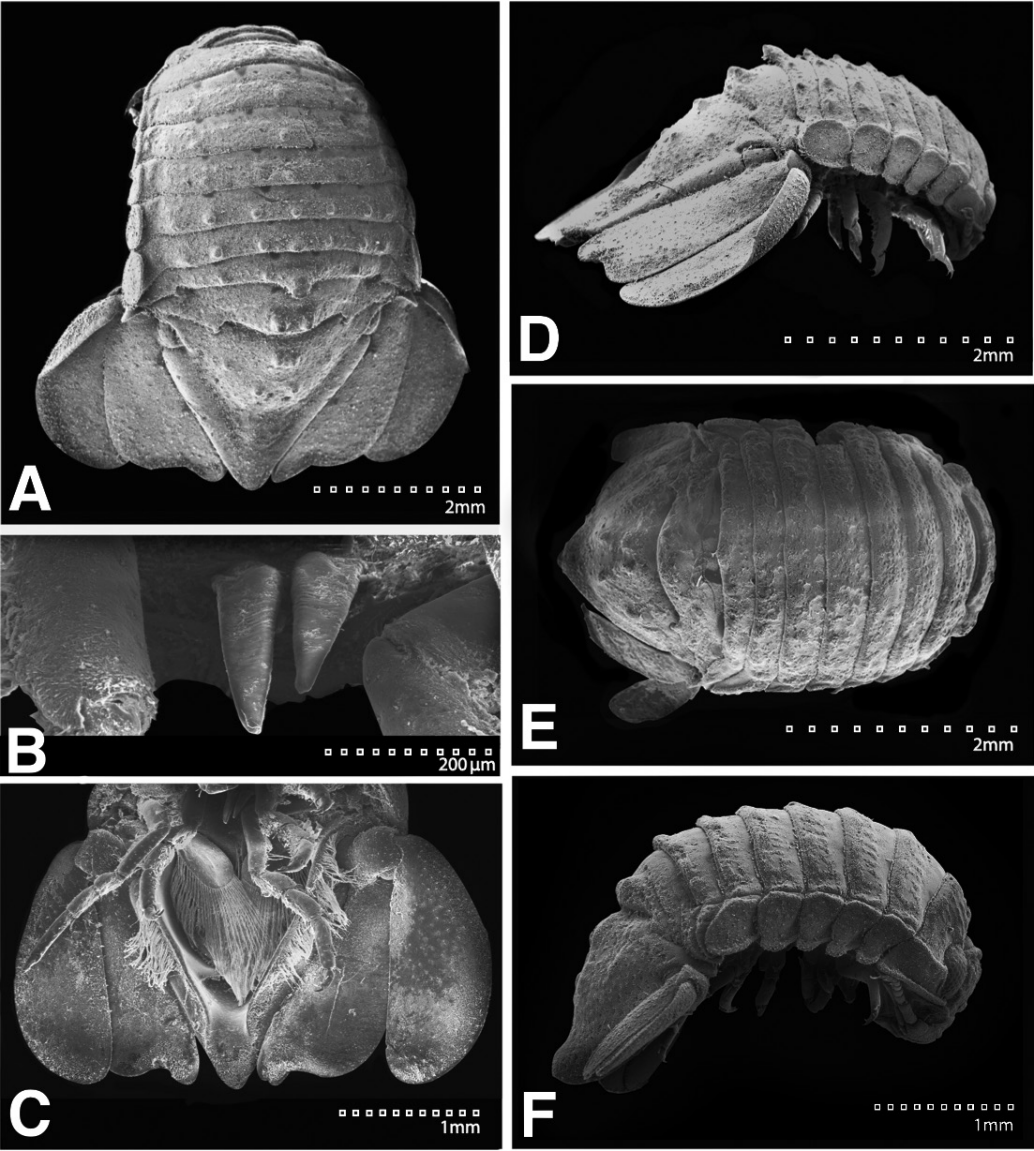
SEMs of *Exosphaeroma amplicauda* LACM CR-2014.1.1. **A** male dorsal **B** penes **C** male pleotelson ventral **D** male lateral **E** female dorsal **F** female lateral.

**Figure 22.**
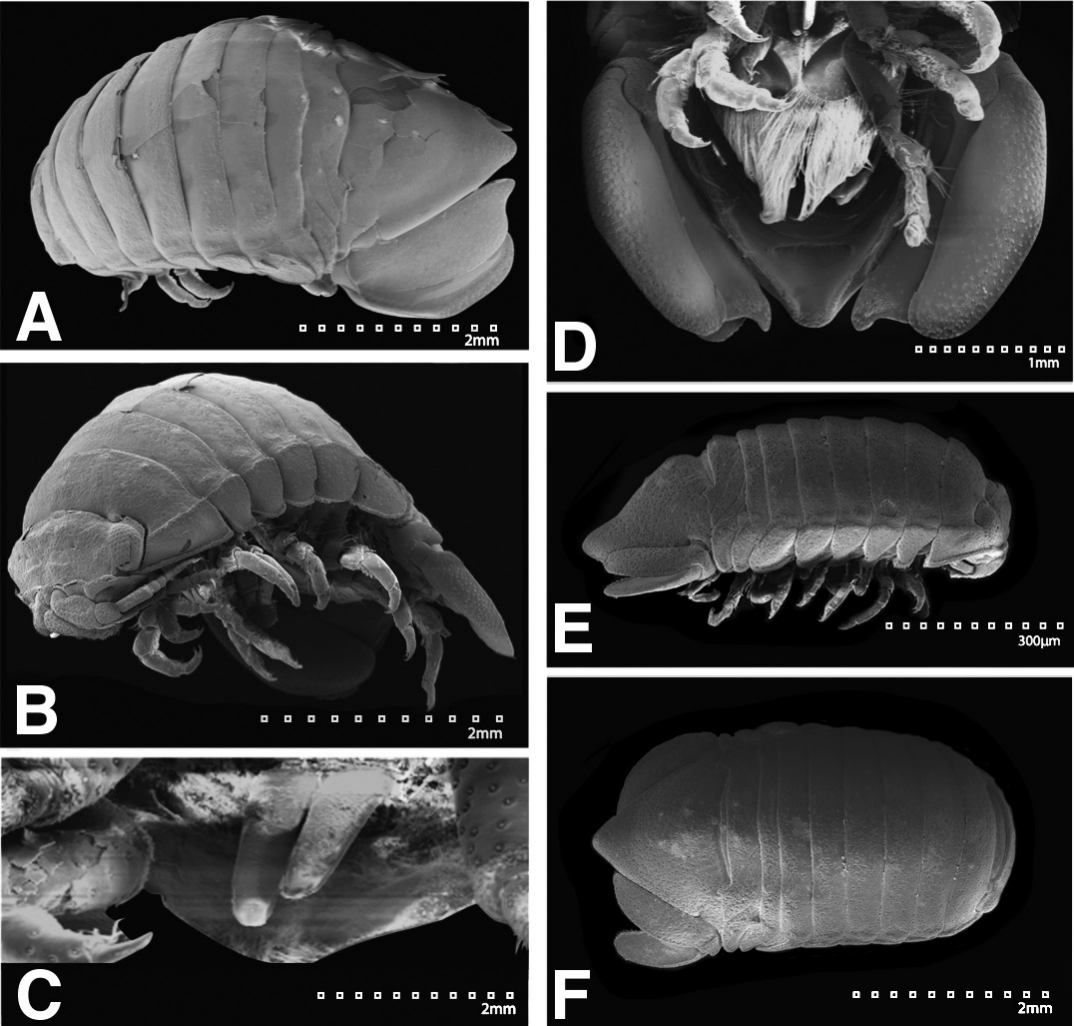
SEMs of *Exosphaeroma paydenae* sp. n. paratype USNM 20474. **A** USNM 20474x male dorsal **B** male lateral **C** penes **D** male pleotelson ventral **E** USNM 20474xi female lateral **F** female dorsal.

**Figure 23.**
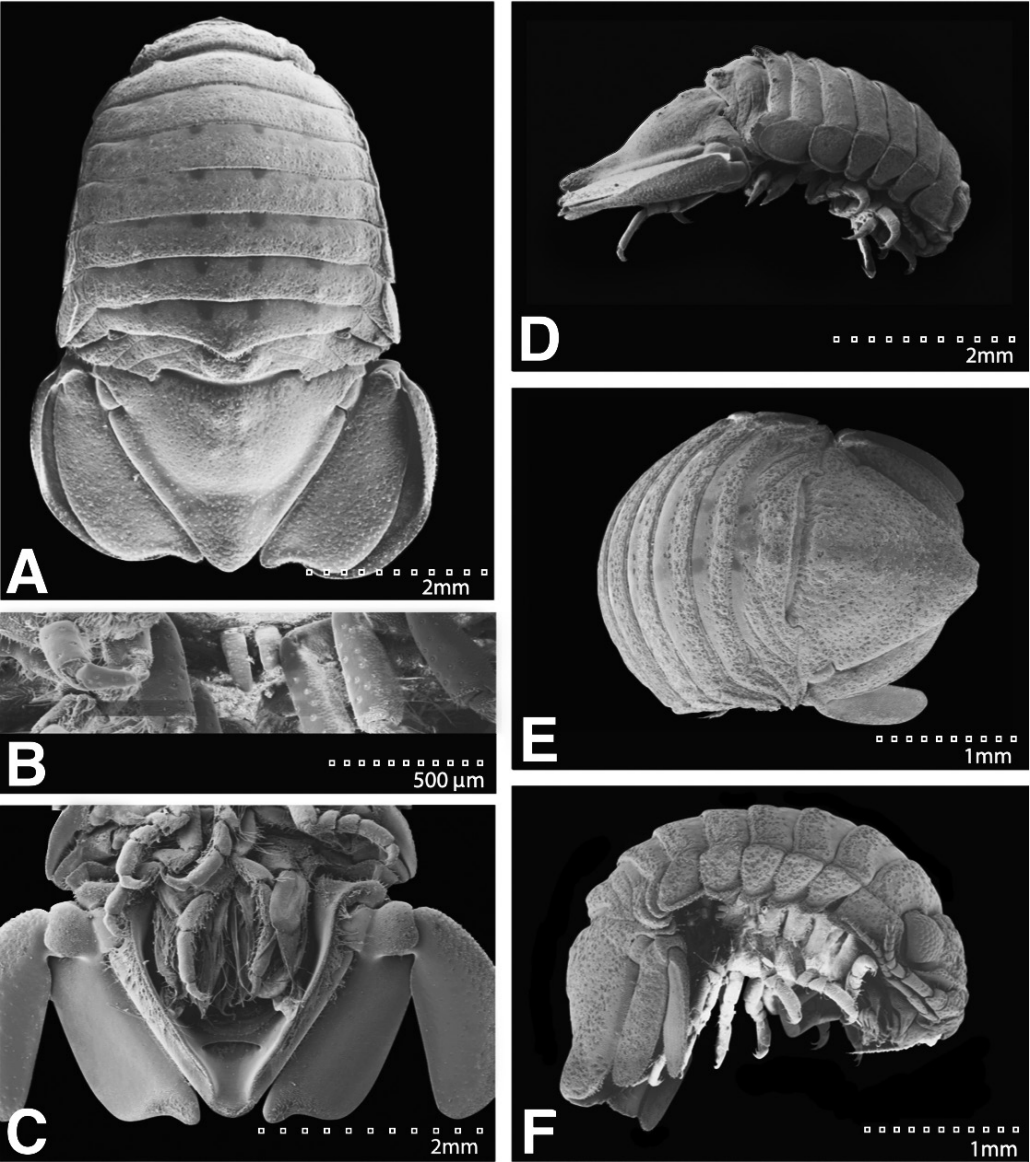
SEMs of *Exosphaeroma russellhansoni* sp. n., paratype. LACM CR-2014.6.4. **A** male dorsal **B** penes **C** male pleotelson ventral **D** male lateral **E** LACM CR-2014.6.5 female dorsal **F** female lateral.

**Figure 24.**
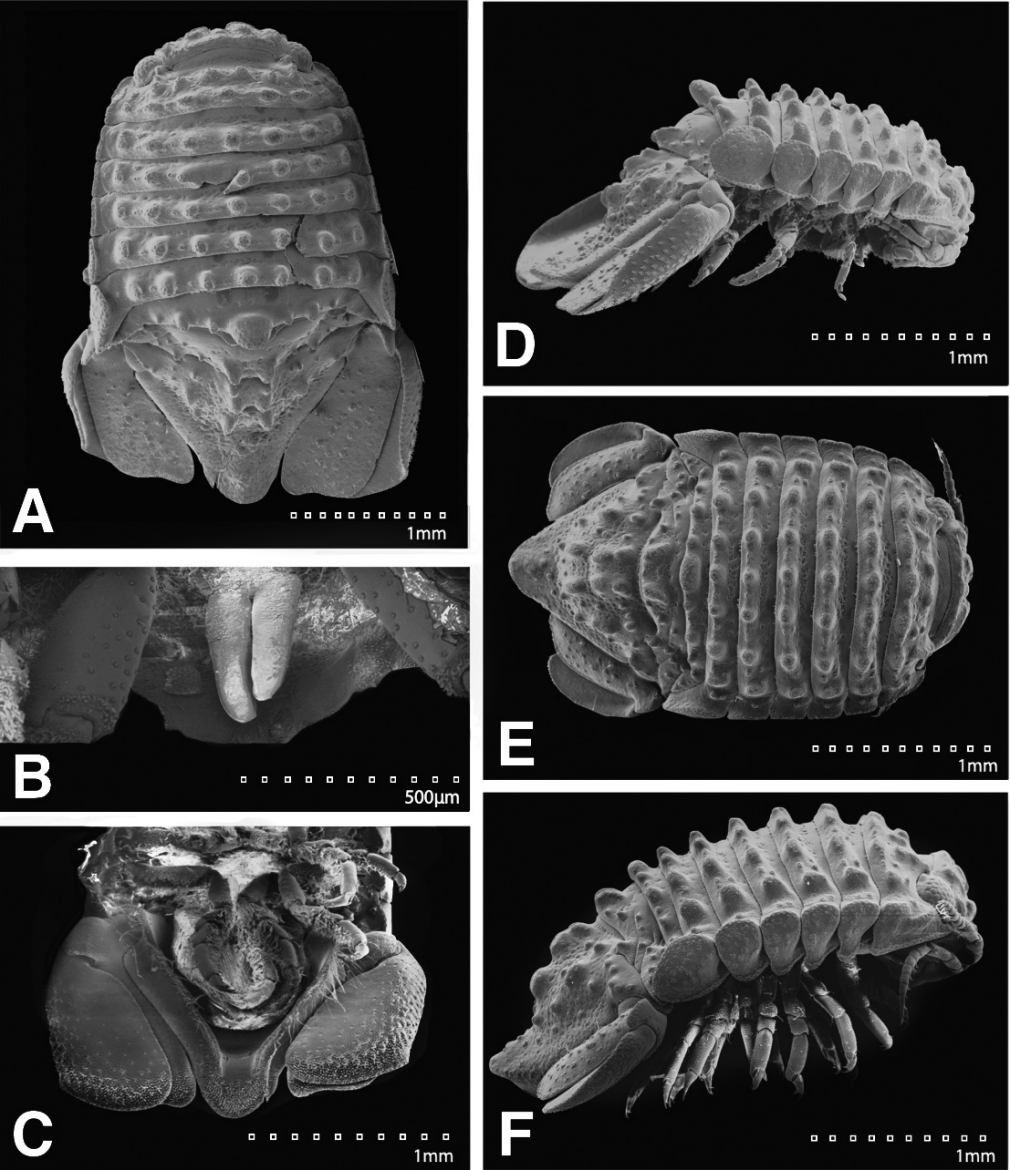
SEMs of *Exosphaeroma pentcheffi* sp. n. paratype LACM CR-2014.12. **A** male dorsal **B** penes **C** male pleotelson ventral **D** male lateral; LACM CR-2014.11 **E** female dorsal **F** female lateral.

**Figure 25.**
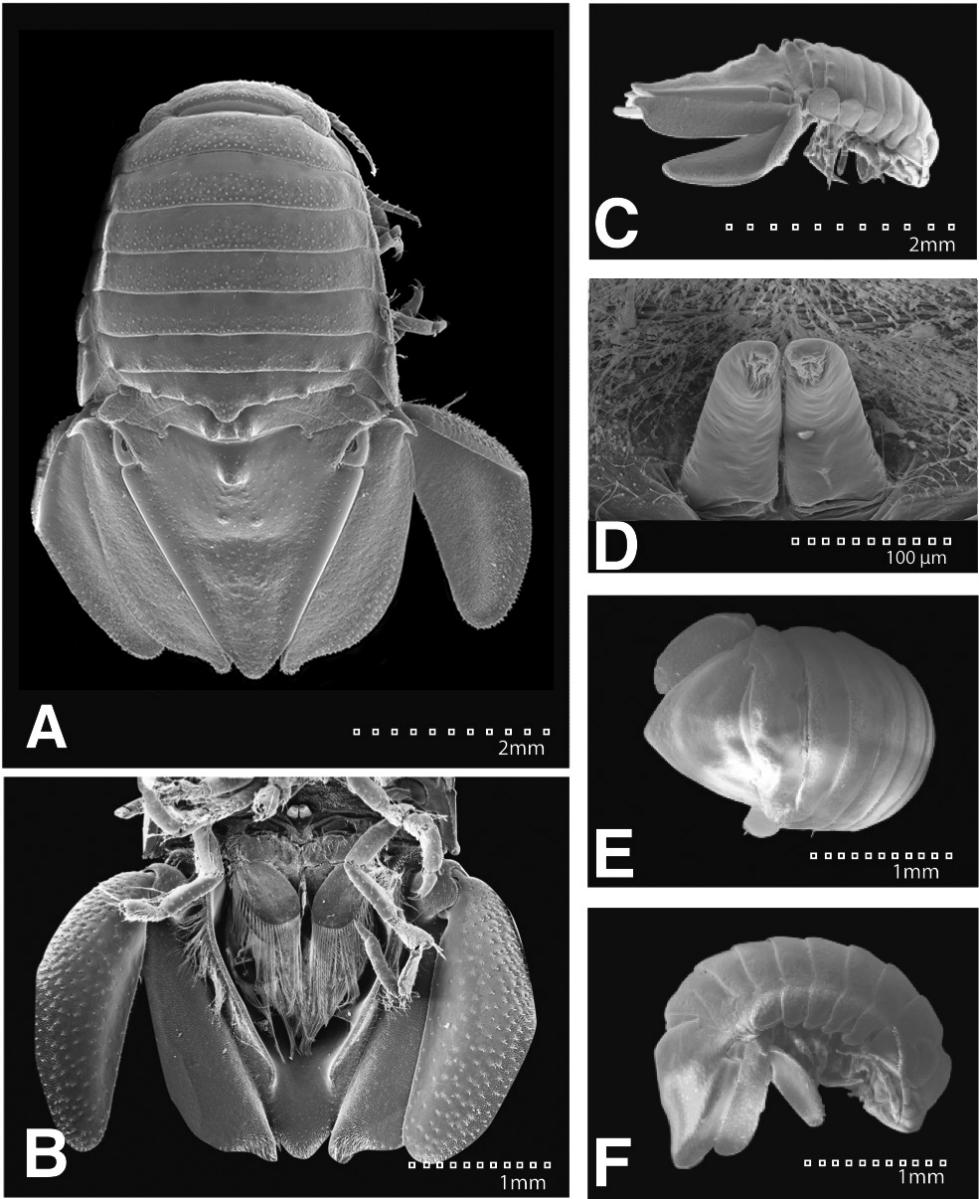
SEMs of *Exosphaeroma aphrodita* LACM CR-2014.14. **A** male dorsal **B** male pleotelson ventral **C** male lateral **D** penes **E** female dorsal **F** female lateral.

**Figure 26.**
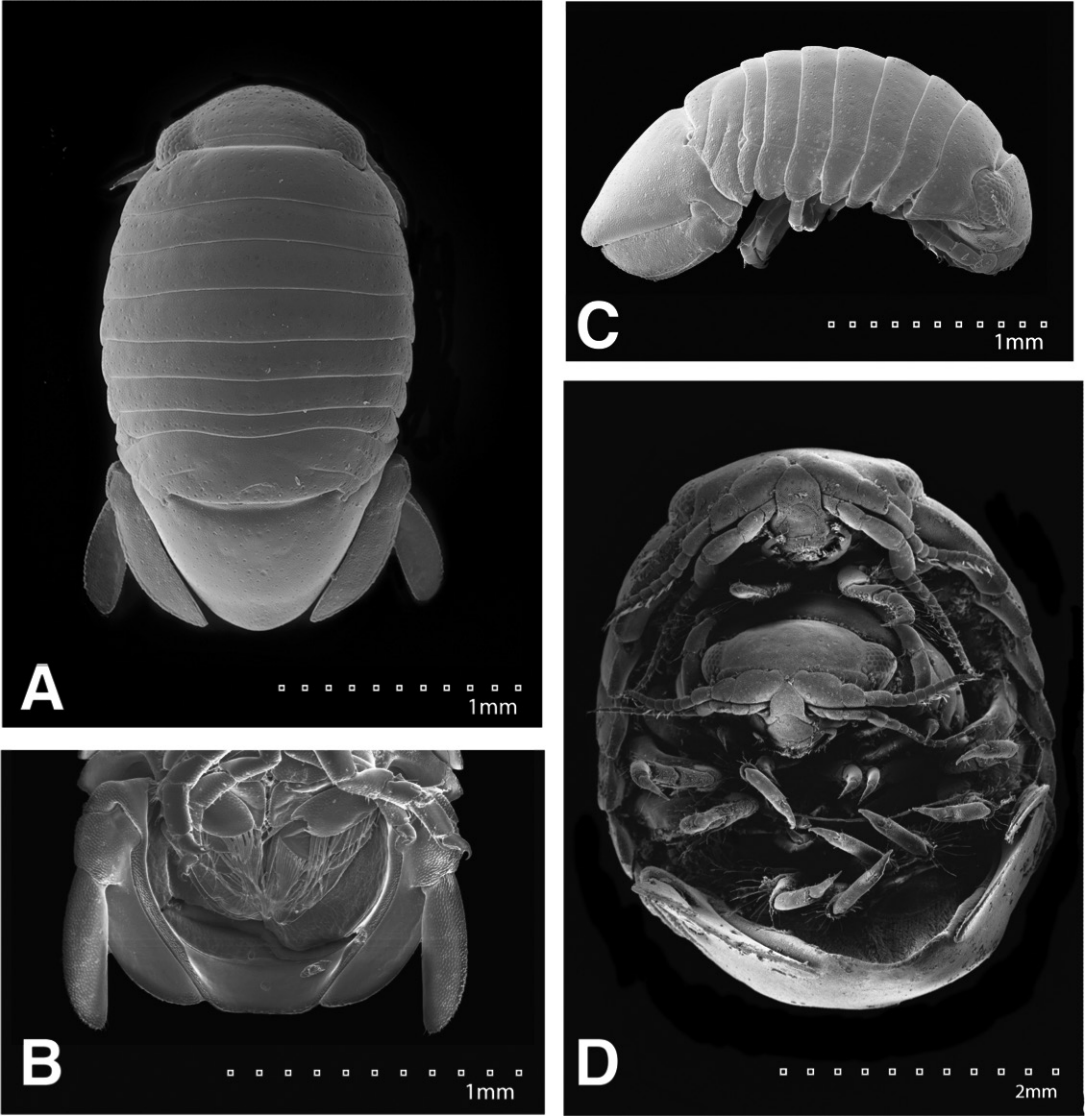
*Exosphaeroma inornata* male RW05.315, USA, California, Los Angeles County, Palos Verdes Peninsula, San Pedro, Pt. Fermin, shore at Paseo del Mar, ~0.5 mi. W of Gaffey Street, 33.71°N, 118.3°W. **A** male dorsal **B** male pleotelson ventral **C** male lateral **D** RW05.106, Pacific, Mexico, Baja California Norte, west of El Rosario, south of Bocana el Rosario, north of Punta Baja, 30.013°N, 115.797°W, male mate-guarding female (male uropods were removed).

**Figure 27.**
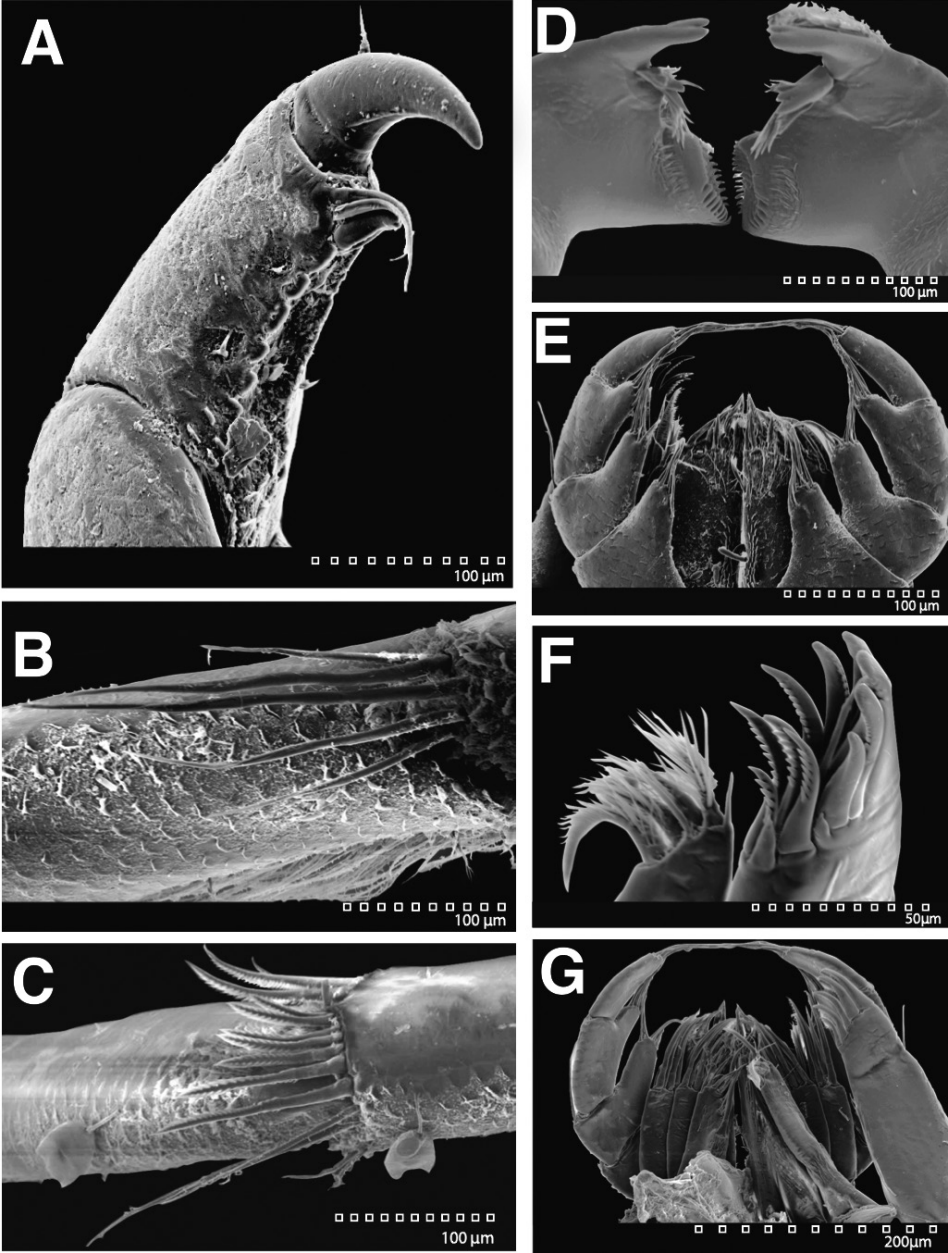
SEM images of *Exosphaeroma amplicauda* LACM CR-2014.1.1. **A** pereopod 3 dactylus scales **B** pereopod 7 merus distal setal patch **C** pereopod 7 carpus distal setal patch **D** left and right mandibles ventral **E** left and right maxillipeds, maxillulae, and maxillae dorsal **F** left maxillula **G** maxilliped and other mouth parts dorsal.

**Figure 28.**
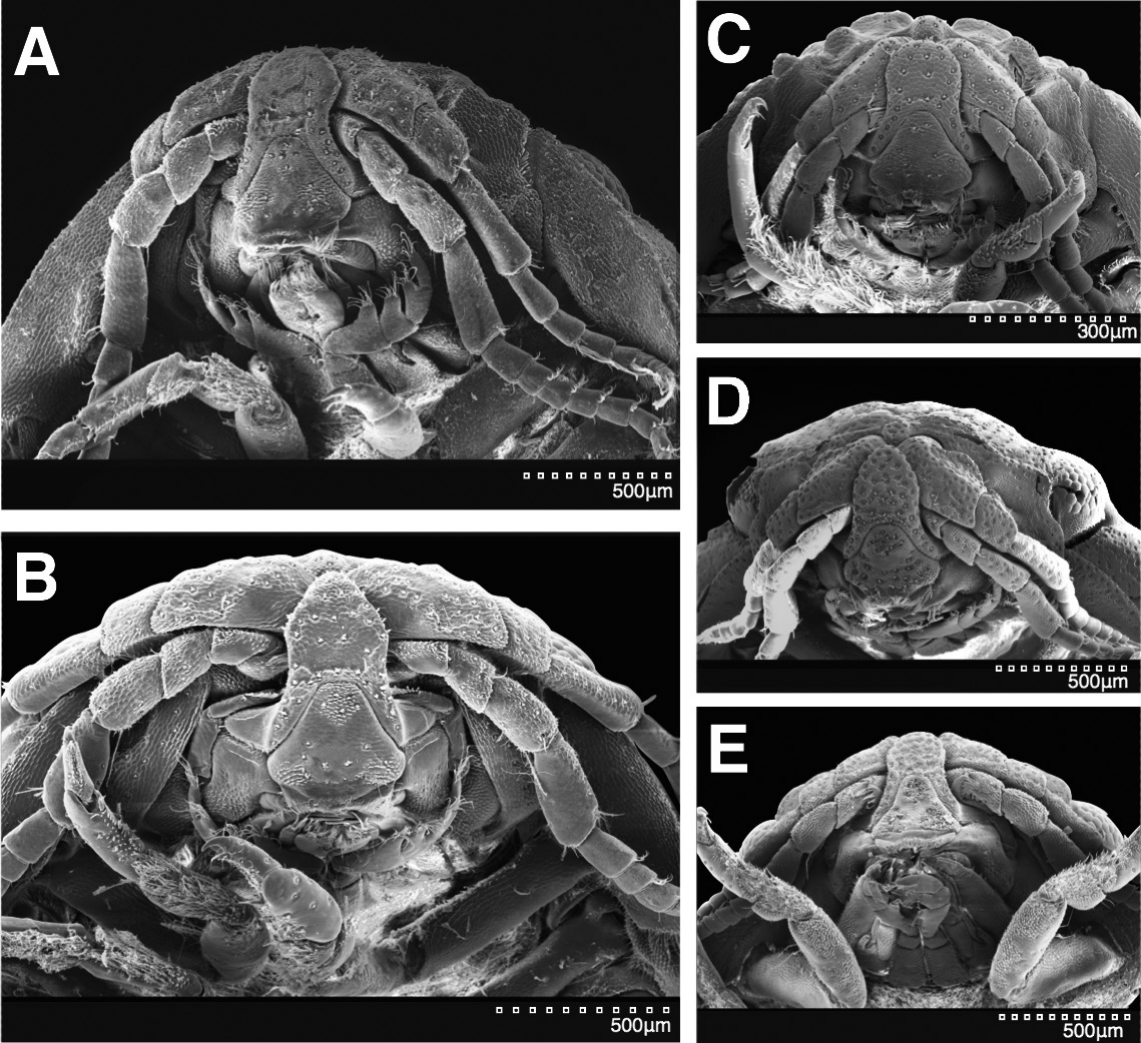
SEM images of epistomes. **A** *Exosphaeroma amplicauda* LACM CR-2014.1.1 **B** *Exosphaeroma aphrodita* LACM CR-2014.14 **C** *Exosphaeroma pentcheffi* sp. n. LACM CR-2014.12 **D** *Exosphaeroma russellhansoni* sp. n. LACM CR-2014.6.4 **E** *Exosphaeroma paydenae* sp. n. USNM 1251663.

## Supplementary Material

XML Treatment for Exosphaeroma

XML Treatment for Exosphaeroma amplicauda

XML Treatment for Exosphaeroma paydenae

XML Treatment for Exosphaeroma russellhansoni

XML Treatment for Exosphaeroma pentcheffi

XML Treatment for Exosphaeroma aphrodita

## Appendix 1

**Sphaeromatidae of the western coast of North America (Alaska to Mexico)**

***Cassidinidea* Hansen, 1905**

*Cassidinidea mexicana* Hendrickx & Espinoza-Pérez, 1998. Gulf of California, Mexico.

***Dynoides* Barnard, 1914**

*Dynoides crenulatus* Carvacho & Haasmann, 1984. Pacific Mexico.

*Dynoides dentisinus* Shen, 1929. Introduced. Kussakin 1979; Kussakin and Malyutina 1993.

*Dynoides elegans* (Boone, 1923) [formerly *Clianella elegans*Li (2000)]. California.

*Dynoides saldani* Carvacho & Haasmann, 1984. Pacific Mexico (SCAMIT 2013).

**“*Dynamene*”**

*Dynamene benedicti* Richardson, 1899. Monterey Bay, California. Correct generic placement uncertain; Kussakin 1979; excluded from the genus by Harrison and Holdich (1982); Brusca et al. 2007.

*Dynamene dilatata* Richardson, 1899; Monterey Bay, California. Correct generic placement uncertain; Kussakin 1979; Brusca et al. 2007.

*Dynamene glabra* Richardson, 1899. Oregon to California. Correct generic placement uncertain; Kussakin 1979; excluded from the genus by Harrison and Holdich 1982; SCAMIT 2013.

*Dynamene sheareri* Hatch, 1947. Oregon. Correct generic placement uncertain; redescribed by George and Strömberg (1968); excluded from the genus by Harrison and Holdich 1982; Brusca et al. 2007 (SCAMIT 2013).

*Dynamene tuberculosa* Richardson, 1899. California. Correct generic placement uncertain; SCAMIT 2013.

***Dynamenella* Hansen, 1905**

*Dynamenella conica* (Boone, 1923). California. Correct generic placement uncertain.

***Exosphaeroma* Stebbing, 1900**

*Exosphaeroma amplicauda* (Stimpson, 1857); = *Exosphaeroma octoncum* (Richardson, 1897). Marin County to Monterey, California. Brusca et al. 2007; SCAMIT 2013.

*Exosphaeroma aphrodita* Boone, 1923. San Diego, California.

*Exosphaeroma bruscai* Espinosa-Perez & Hendrickx, 2002. Pacific Mexico.

*Exosphaeroma inornata* Dow, 1958; = *Exosphaeroma media* George & Strömberg, 1968. Puget Sound, Washington to Southern California. Iverson 1978, 1982; Brusca et al. 2007.

*Exosphaeroma paydenae***sp. n.** Alaska.

*Exosphaeroma pentcheffi***sp. n.** Los Angeles, California.

*Exosphaeroma rhomburum* (Richardson, 1899); Monterey Bay, California. *Incertae sedis*, males not known.

*Exosphaeroma russelhansoni***sp. n.** Washington.

***Gnorimosphaeroma* Menzies, 1954**

*Gnorimosphaeroma insulare* (Van Name, 1940), Washington. Menzies 1954; Hoestlandt 1977; Brusca et al. 2007.

*Gnorimosphaeroma noblei* Menzies, 1954. Washington to California. Kussakin 1979; Brusca et al. 2007.

*Gnorimosphaeroma oregonense* (Dana, 1852). Alaska to northern California. Menzies 1954; Kussakin 1979, Brusca et al. 2007, SCAMIT 2013.

*Gnorimosphaeroma rayi* Hoestland, 1969. Nunomura 1998; Brusca et al. 2007.

***Paracerceis* Hansen, 1905**

*Paracerceis cordata* (Richardson, 1899). California. Kussakin 1979; Brusca et al. 2007.

*Paracerceis gilliana* (Richardson, 1899); California.

*Paracerceis granulosa* (Richardson, 1899); Cerros Island, California.

*Paracerceis richardsoni* Lombardo, 1988. Pacific Mexico. Espinosa-Pérez and Hendrickx 2002.

*Paracerceis sculpta* (Holmes, 1904). San Clemente Island, California. Globally translocated and widespread. Menzies 1962; Harrison and Holdich 1982; Nunomura 1988; Loyola e Silva et al. 1999; Brusca et al. 2007.

*Paracerceis spinulosa* Espinosa-Perez & Hendrickx, 2002. Sonora, Mexico.

***Paradella* Harrison & Holdich, 1982**

*Paradella dianae* (Menzies, 1962). Brusca et al. 2007.

*Paradella tiffany* Bruce & Wetzer, 2004; Baja California, Mexico.

*Paradella garsonrum* Wetzer & Bruce, 2007; Baja California, Mexico.

***Pseudosphaeroma* Chilton, 1909**

Pseudosphaeroma sp. cf. campbellensis Bruce & Wetzer, 2008. Introduced species San Francisco to Morro Bay, California. Not *Pseudosphaeroma campbellensis* of Hurley and Jansen (1977) and Harrison (1984).

***Sphaeroma* Latreille, 1802**

*Sphaeroma quoianum* Milne Edwards, 1840 [= *Sphaeroma pentodon* Richardson, 1904]. Introduced to San Francisco and San Diego, California. Baker 1926; Hale 1929; Kussakin 1979; Harrison and Holdich 1984; Brusca et al. 2007; (SCAMIT 2013).

*Sphaeroma serratum* (Fabricius, 1787). Worldwide. Kussakin 1979; Jacobs 1987.

*Sphaeroma walkeri* Stebbing, 1905. Introduced to San Diego, California. Carlton and Iverson 1981; Pires 1982; Brusca et al. 2007, Kussakin 1979.

***Striella* Glynn, 1968**

*Striella* sp. La Paz, Gulf of California (LACM collections, UC Mexus station 43).
